# Interventions promoting resilience through climate smart agricultural practices for women farmers: A systematic review

**DOI:** 10.1002/cl2.1426

**Published:** 2024-08-27

**Authors:** Ashrita Saran, Sabina Singh, Neha Gupta, Sujata Chodankar Walke, Ranjana Rao, Christine Simiyu, Suchi Malhotra, Avni Mishra, Ranjitha Puskur, Edoardo Masset, Howard White, Hugh Sharma Waddington

**Affiliations:** ^1^ Campbell Collaboration, South Asia Delhi India; ^2^ Delhi New Delhi India; ^3^ Campbell South Asia Delhi New Delhi India; ^4^ Campbell South Asia Delhi India; ^5^ London School of Hygiene and Tropical Medicine London UK; ^6^ Campbell Collaboration Vasant Kunj India

**Keywords:** climate‐smart agriculture (CSA), gender, knowledge dissemination approaches, sustainable agriculture, women farmers

## Abstract

**Background:**

Climate change poses a significant threat to agricultural production worldwide, with developing countries being particularly vulnerable to its negative impacts. Agriculture, which is a crucial factor in ensuring food security and livelihoods, is particularly vulnerable to changes in climate patterns, such as increased temperatures, drought, and extreme weather events. One approach to addressing these challenges is by promoting the adoption of climate‐smart agriculture (CSA) practices among farmers. CSA combines traditional agricultural practices with innovative techniques and technologies to adapt to and mitigate the impacts of climate change. infrastructure. By adopting CSA practices, farmers can enhance their resilience to climate variability and improve their productivity.

**Objectives:**

This review examines the effectiveness of interventions promoting CSA to enhance farmers' knowledge of the benefits of CSA approaches, subsequent adoption of CSA, and disadoption of harmful agricultural practices in low‐ and middle‐income countries (LMICs).

**Search Methods:**

We searched 39 academic and online databases, websites, and repositories and screened over 19,000 experimental and quasi‐experimental publications to identify studies promoting CSA practices to women farmers. We conducted a citation tracking process on included studies and contacted experts to ensure a thorough search.

**Selection Criteria:**

The review focused on studies that included interventions promoting climate‐smart agricultural approaches. Using EPPI Reviewer 4, two review authors independently screened the impact evaluation using a standardized screening tool.

**Data Collection and Analysis:**

Information about participant characteristics, intervention characteristics, control conditions, research design, sample size, bias risk, outcomes, and results were gathered. Data collection and quantitative analysis were conducted using standard Campbell Collaboration methods.

**Main Results:**

Eight impact evaluations were found (two randomized controlled trials) evaluating the effects of CSA practices on farmer's knowledge gains of the benefits of CSA practices and subsequent adoption. Knowledge dissemination approaches such as Farmer Field Schools and weather and climate information services were found to positively impact farmers' knowledge and adoption of specific CSA practices. However, the evidence supporting this claim is uncertain as a high risk of bias was assessed for five of the eight included studies. However, we found no effects on the disadoption of harmful practices such as pesticide overuse.

**Authors' Conclusions:**

The evidence base for studies promoting climate‐smart agricultural approaches (CSA) to farmers in LMICs is small, and there is a lack of studies reporting sex‐disaggregated data and studies explicitly targeting women farmers. The review suggests that knowledge dissemination techniques are significantly effective in improving CSA knowledge and adoption, including integrated pest management techniques and their benefits, adoption of climate‐resilient rice seed varieties (STRVs), and use of botanical pesticides by farmers. More and better confidence studies are needed to inform policy and programming, including those that look at a wider range of interventions, including changing norms, values, and institutional arrangements.

## 1 PLAIN LANGUAGE SUMMARY

Promoting climate‐smart agricultural practices improves smallholder farmers' knowledge and farming practices in low‐ and middle‐income countries (LMICs), but it is limited in scope and methodological rigor.

### 1.1 The review in brief

Interventions promoting climate‐smart agriculture (CSA) approaches, such as farmer field schools and agriculture extension services, show improvements in knowledge gains and adoption of these practices by smallholder farmers in LMICs. However, due to methodological limitations, confidence in study findings is low.

### 1.2 What is this review about?

Climate change has been seen to cause extreme weather conditions and natural disasters such as heavy rainfall, drought, catastrophic storms, pest infestation, wildfires, flooding, and declining biodiversity, which in turn reduces global food production and security, particularly in developing countries. Women, who make up approximately 43% of the agricultural labor force, are the most affected by climate change threats due to their disadvantaged social, economic, and cultural position. For example, female farmers in developing nations have less access to modern technology, markets, information, assets, and credit than their male counterparts. CSA aims to prevent climate change and food insecurity by providing agricultural methods that increase production and income without disrupting the ecosystem and natural resources. Therefore, by introducing women farmers to CSA, we can improve their resilience to climate change effects by providing timely access to valuable information and user‐friendly technology.

### 1.3 What is the aim of this review?

We conducted a systematic review of interventions aimed at promoting CSA practices in LMICs, with a focus on enhancing women farmers' knowledge and adoption of CSA while discouraging harmful agricultural practices.

### 1.4 What were the main findings of this review?

The review analyzed eight impact evaluations that assessed the effectiveness of CSA promotion interventions. All of these interventions were knowledge dissemination approaches. All the included studies examined the impact of the intervention on both women and men farmers. However, studies explicitly targeting women farmers or providing sex‐disaggregated data were lacking.

Four studies examined how farmer field schools, or their modification, could increase farmers' knowledge about the benefits of CSA practices and their subsequent adoption. Only one study, which used participatory action research and awareness campaigns, successfully increased knowledge and led to the trial adoption of water‐saving technologies among farmers in rural areas. In contrast, two studies that used video‐assisted learning on botanical pesticide use and climate‐resilient rice seed varieties found positive changes in farmers' knowledge scores and subsequent adoption. Only one study aimed to incorporate a gender‐focused approach by ensuring equal participation and representation of women in the training sessions. Another study investigated the link between weather and climate information services and the adoption of multiple cropping practices and water management and found that the use of weather and climate information services significantly increases the adoption of water management and multiple cropping practices.

Knowledge dissemination approaches were found to positively impact farmers' knowledge and adoption of specific CSA practices. However, the evidence supporting this claim is uncertain. Significant gaps exist in sex‐disaggregated data and crucial interventions, such as studies on financial incentives and institutional arrangements, which are essential in improving the adoption of CSA approaches.

### 1.5 What do the findings of this review mean?

These review findings imply that while knowledge dissemination approaches promise to advance CSA practices among farmers, the evidence is not robust across all dimensions. More research is needed, mainly to understand the role of gender and the influence of financial and institutional factors in promoting and adopting CSA practices.

### 1.6 How up‐to‐date is this review?

The review authors searched for studies published between January 2010 and February 2022.

## 2 SUMMARY OF FINDINGS

Summary of findings 1

Intervention characteristics of selected studies.

**Table:** 

Type of response to climate change	Examples of specific activities associated with each response to climate change	Type of promotional interventions	Studies reporting responses to climate change
Climate‐resilient crop seeds	Stress tolerant rice variety (STRV)	Quality seed production training (QSP)	Dar et al. (2020)
Use of fertilizers and pesticides	Agronomic practices	Weather and climate information services (weather forecasts, call centers, agro‐advisories, input and output market prices) Farmer field schools Video‐mediated learning	Djido et al. (2021) Guo et al. (2015); Mancini et al. (2006) Chowdhury et al. (2015)
Modified planting activities	Multiple cropping practices	Weather and climate information services (weather forecasts, call centers, agro‐advisories, input and output market prices)	Djido et al. (2021); Nyasimi (2007)
Irrigation and water management	Water conservation strategies, irrigation, micro irrigation, water harvesting and improving drainage	Weather and climate information services (weather forecasts, input and output market prices)	Djido et al. (2021); Sharma et al. (2019); Osumba et al. (2021)
Carbon or nutrient management	Nutrient management, agro‐environment and cultivation	Farmer field schools	Guo et al. (2015)

Summary of findings 2

Intervention content and implementation details.

**Table:** 

Study	Country	Study type	Sample size	Intervention type	Intervention description	Setting
Chowdhury et al. (2015)	Bangladesh	Controlled trial	288 farmers; 151 males and 137 females	Knowledge dissemination and capacity building approaches & intervention to promote participation in CSA activities	Video and discussion: Botanical pesticide innovation through video mediated learning	16 villages in 3 sub‐districts (Sajahanpur, Sherpur and Bogra Sadar)
Dar et al. (2020)	India	Randomized controlled trial	1220 farmers, 610 female farmer and 610 male farmers were selected from 61 villages.	Knowledge dissemination and capacity building approaches & intervention to promote participation	Training and inputs: Farmers receive Quality Seed Production (QSP) trainings, access to climate‐resilient rice seed variety and agro‐based goods (IRRI‐bags)	61 villages were selected from 34 districts in Eastern Indian states of Bihar, Chhattisgarh, Jharkhand, Odisha and West Bengal. The areas targeted represent the abiotic stress prone ecologies mainly affected by drought.
Djido et al. (2021)	Ghana	Bi‐variate probit model	900 farmers	Knowledge dissemination and capacity building approaches & intervention to promote participation	Weather information services: Farmers received weather and climate information services (WCIS) under the project by CGIAR Research Program on Climate Change, Agriculture and Food Security (CCAFS).	Data was collected from the upper West Region of Ghana including Tabier, Orbili, Tuori, Bompari, Dazuuri, Wulling, Die, Jeffiri, Baazu, and Doggoh
Guo et al. (2015)	China	Randomized controlled trial	711 households, 299 female and 412 males.	Knowledge dissemination and capacity building approaches & intervention to promote participation	Training: Agriculture extensions and skill training through Farmer Field Schools	Anhui Province in two countries Chaohu and Tianchang
Mancini et al. (2006)	India		137 smallholder farmers	Knowledge dissemination and capacity building approaches & intervention to promote participation	Training: Integrated pest management training	
Nyasimi et al. (2017)	Tanzania	Cross‐sectional	81 households; 54 male‐headed and 27 females headed	Knowledge dissemination and capacity building approaches & intervention to promote participation	In this farmer were exposed to the Farms of the Future (FotF), climate‐analog tool developed by Consultative Group for International Agricultural Research (CGIAR) Research Program on Climate Change, Agriculture and Food Security (CCAFS)	Two villages Yamba and Mbuzii were selected from Lushoto, Norther Tanzania
Osumba et al. (2021)	Kenya, Tanzania and Uganda	Cross‐sectional	661 participants, 209 females and 452 males and 36 agri‐business	Knowledge dissemination and capacity building approaches & intervention to promote participation	Climate‐resilient agribusiness FFS (CRAFFS)	Kenya, Tanzania and Uganda
Sharma et al. (2019)	India	Pre‐and‐post	150 farmers, 75 female farmers and 75 male farmers	Knowledge dissemination and capacity building approaches & intervention to promote participation	Awareness campaigns and street plays on water saving technologies	Ropar, Ludhiana and Faridkot districts were selected from the three zones of Punjab namely north‐east zone, central zone and south‐west zone. One village with the highest rate of groundwater depletion and paddy–wheat crop rotation was selected from each of the selected districts, namely, Sandhua (Ropar), Talwandi Khurd (Ludhiana) and Ransingh Wala (Faridkot)

Summary of findings 3

Summary of findings.

**Table:** 

Outcome	Studies reporting outcomes	Effect	Summary
Knowledge	Chowdhury et al. (2015), Dar et al. (2020), Guo et al. (2015), Mancini (2006)	*d* = 0.30 (0.13 to 0.46) *n* = 4 *I*² = 75% Egger's test −3.47 (*t* = −2.15, *p* = 0.16)	Moderate effect based on small number of studies with moderate heterogeneity and small publication bias
Disadoption	Chowdhary et al. (2015) Mancini (2006)	*d* = 0.21 (0.08 to 0.50) *n* = 2 *I*² = 80% Eggers test Too small sample size	Small effect based on small number of studies with high heterogeneity and potential publication bias
Adoption	Chowdhary et al. (2015), Dar et al. (2020), Mancini (2006)	*d* = 0.25 (0.03 to 0.48) *n* = 3 *I*² = 81% Eggers test Too small sample size	Small effect based on the low number of studies with high heterogeneity and potential publication bias

Summary of findings 4

Outcome variable categories.

**Table:** 

Outcome type	Examples of outcome sub‐types included	Studies reporting outcome
Knowledge	Knowledge score (pest management, nutrient management, decision making), knowledge of helpful practices (using plants as pesticide), knowledge of harms (chemical pesticides)	Chowdhury et al. (2015), Guo et al. (2015), Mancini (2006)
Attitudes	Agree with beneficial practices	Chowdhury et al. (2015)
Disadoption (unfavoured practice)	Number of sprays during first 40 days, use and cost of pesticide	
Adoption (favored practice)	Adoption of CSA, use of botanical inputs, safe storage of inputs	Chowdhury et al. (2015), Dar et al. (2020), Djido et al. (2021), Mancini (2006)
Management	Sustainable farm	Guo et al. (2015)
Time use	Total labor use, labor use for specific tasks (e.g. pesticide, IPM tasks), female share of total work	Mancini (2006)
Yield	Kilogrammes per hectare	Mancini (2006)

## 3 BACKGROUND

### 3.1 The problem, condition or issue

Climate change is projected to have a substantial and widespread impact on global agricultural production, food security and livelihoods, and developing countries are highly susceptible to further negative consequences (IPCC, 2021). Extreme climate change events, such as droughts, heavy rainfall, flooding, water scarcity, severe fires, rising sea levels, melting polar ice, catastrophic storms, and declining biodiversity, will accelerate in many regions worldwide, influencing food production and supply (Hasegawa et al., 2021). The average and seasonal maximum temperatures will continue to rise, threatening crops, wildlife, and freshwater supplies. High CO_2_ levels can affect crop yields and are associated with reduced protein and nitrogen content in most crops, such as wheat, rice, barley, oats, and potatoes, resulting in quality loss (USGCRP, 2018). Reduced grain quality could affect livestock, which contributes more than 15% of the global human protein supply (USGCRP, 2018). Heat stress further increases livestock vulnerability to diseases and reduces livestock fertility and milk production. Moreover, moisture‐reliant pathogens can thrive in areas with high rainfall. An increase in the use of parasiticides may cause parasiticides to enter food chains (USGCRP, 2018). Rising ocean temperatures and ocean acidification have severely impacted the sustainability of fisheries and aquaculture and, thus, the livelihoods of the communities that depend on fisheries (Portar et al., 2014).

Land use, land cover change (LULCC), and climate change are interrelated. From 2007 to 2016 land use (cultivation of crops and livestock) and land use change caused by deforestation accounted for approximately 13% of CO_2_, 44% of methane (CH_4_), and 81% of nitrous oxide (N_2_O) emissions, representing 23% of the total global greenhouse gas (GHG) emissions (IPCC, 2019). On the other hand, global climate change impacts LULCC by changing precipitation patterns, degrading land, and increasing temperatures (Mbow et al., 2019; IPCC, 2019). The negative impacts of climate change on land include depletion of natural resources, disruption of water cycles, decline in biodiversity, and spread of diet‐induced diseases. Developing countries are particularly vulnerable to climate change and LULCC owing to their geographical and climatic conditions. Climate change has led to issues related to soil and land degradation, including soil acidity, unavailability of micronutrients, low carbon content, and low water‐holding capacity (Elbehri et al., 2017). Increased desertification is estimated to have the greatest impact in Asia and Africa. A growing number of wildfires may affect North America, South America, the Mediterranean, southern Africa, and Central Asia and a decline in crop yields is expected to be most pronounced in the tropics and subtropics (IPCC, 2019). Statistics show that 65% of the workforce in Sub‐Saharan Africa and 60% in South Asia work in agriculture and depend solely on agriculture (covering crops and livestock production, as well as forestry, fisheries, and aquaculture) for income livelihoods, food, and nutrition security (Ado et al., 2019; Hasegawa et al., 2021). Hence, many of the most vulnerable people are exposed to the effects of climate change and LULCC through the loss of rural livelihoods and income, marine and coastal ecosystems, terrestrial and inland water ecosystems, and food insecurity (Mbow et al., 2019).

#### 3.1.1 Why women are vulnerable to climate change?

In agriculture, women are disproportionately affected by the threats and shocks posed by climate change, especially in developing countries (Paavola & Adger, 2006; Petheram et al., 2010; UNDP, 2016). Women account for approximately 43% of the agricultural labor force in developing countries and are essential to supporting production and providing food, nutrition, and income security (Food and Agriculture Organization [FAO], 2012). Women's vulnerability to climate change stems from social, economic and cultural factors. Women comprise 70% of the 1.3 billion impoverished people. Among poor urban households, women head 40% (Munoz Boudet et al., 2018). More than half of the world's food is produced by women (50%–80%), but they own less than 10% of the land (Paudyal et al., 2019). Women also have less access to resources such as land, credit, agricultural inputs, decision‐making structures, technology, training, and extension services, which would enhance their capacity to adapt to climate change (Howland et al., 2019). In most cases, women are responsible for the time‐consuming and labor‐intensive tasks carried out by hand or with simple tools. Subsistence farming is primarily performed by women, who raise poultry, small livestock, and horticulture (Wester & Lama, 2019). Environmental goods and services are only partially accessible and controlled by women; they have little input into decision‐making and do not share the benefits of managing the environment. Lack of access to resources leads women to over rely on harmful coping mechanisms, such as maladaptive agricultural practices, overcropping (which depletes soil fertility), and the use of scarce agricultural inputs (e.g., water and time) for other purposes (Berrang‐Ford et al., 2021; Chanana‐Nag & Aggarwal, 2020). Furthermore, they may simply fail to adapt to changing circumstances to grow crops that suit their household nutritional needs. This makes women less able to cope with climate change than men. Having to travel farther to get water and wood for heating is likely to increase the burden on women in such situations (WHO, 2016). A wide range of gender‐based inequalities affect women living in many developing countries, including human rights, political rights, economic status, land ownership and housing conditions (Huyer & Partey, 2020). Thus, women's vulnerability is exacerbated by climate change. During climate shocks, women are also highly vulnerable to physical, sexual, and domestic violence (Desai & Mandal, 2021). Furthermore, women's coping mechanisms and resilience patterns to climate stressors vary because of the complex interplay between ethnicity, religion, class, and age (Jost et al., 2016). It is evident that women play a critical, potentially transformative role in addressing food insecurity in their households and communities but continue to face obstacles (Nhat Lam Duyen et al., 2021; Paudyal et al., 2019; Teklewold et al., 2019; Tomayko et al., 2017; FAO et al., 2021).

#### 3.1.2 What is climate‐smart agriculture (CSA)

CSA has emerged as a promising way to ensure adequate food supplies for the growing population and to meet the challenges of climate change (Totin et al., 2018; FAO, 2010). The FAO of the United Nations defines CSA as “agriculture that sustainably increases productivity, enhances resilience, reduces greenhouse gases, and enhances achievement of national food security and development goals” (FAO, 2012). CSA aims to address food insecurity and climate change by promoting approaches that increase agricultural production and income without depleting natural resources and vital ecosystems, encouraging resilience and climate change adaptation, and reducing GHG emissions (FAO, 2012). The wide range of technologies and practices in CSA are hereafter referred to as CSA approaches. We used the following classification of CSA approaches, adapted from (Aggarwal et al., 2018) and the (World Bank, FAO and IFAD, 2015), to guide our study inclusion criteria and draft and implement the search.

- water smart: water harvesting and water management and community management of water
- weather smart: weather warning systems, agro‐advisors, and weather insurance
- seed/breed smart: high‐yield, stress‐tolerant seed varieties
- carbon/nutrient smart: cover cropping; efficient fertilizer usage; no‐till or minimum‐tillage farming
- institutional/market smart: financial services, market information, off‐farm risk management, gender strategies, and cross‐sector linking

Previous studies show which CSA approaches work best and where, why, and how they perform well (FAO and Care, 2019; Aggarwal et al., 2018). However, evidence is lacking regarding whether CSA approaches are available, accessible, and able to make a difference for smallholder farmers (both men and women) (Totin et al., 2018; Schut et al., 2016 Rosenstock et al., 2016). Additionally, there is a lack of evidence on whether these interventions have the potential to make a meaningful difference in empowering and enhancing women resilience. To bridge this gap, we conducted a systematic review of the literature and synthesized the existing evidence on interventions used to promote CSA among smallholder women farmers in LMICs. The objective of this study was to identify and evaluate the effectiveness of these interventions, focusing on their ability to empower women farmers and contribute to their resilience in the face of challenges associated with traditional agricultural practices.

### 3.2 The intervention

This review includes studies on interventions that may be used to support the adoption of CSA approaches by smallholder farmers, not the impact of CSA approaches per se (Lopez‐Avila et al., 2017):

- knowledge dissemination approaches, such as Farmer Field Schools (FFS) and peer learning (e.g., local champions), information and communication technologies (e.g., telephone, text, radio, and television), and group and individual training and demonstration (e.g., extensions, demonstration plots, field days, and schools)
- financial approaches, including credit and subsidies (e.g., cash transfers, vouchers, and matching grants), insurance against loss, and advice on risk management
- institutional arrangements, including collectivization (e.g., farmer cooperatives and federations), contract farming, land titling, and community infrastructure (e.g., irrigation dams)

### 3.3 Potential implementation of CSA approaches

The adoption of CSA approaches is based on the understanding that farmers see an advantage in following or are incentivized to follow these agricultural practices instead of their conventional ones (FAO, 2014; Khatri‐Chhetri et al., 2020). These interventions may be based on various underlying principles and approaches. Interventions at one end of the spectrum follow a top‐down approach; for example, FFS are a form of agricultural extension service where experts train groups of farmers on improved agricultural techniques. The FFS approach aims to educate farmers about specific practices such as pest management, crop rotation, and the use of improved seed varieties to enhance productivity and sustainability (Sivabalan et al., 2021). At the other end of the spectrum are interventions built on local synergies and following participatory (bottom‐up). Participatory Action Research in agriculture involves farmers and researchers working together to identify problems, develop solutions, and implement practices that are suited to local conditions (Lacombe et al., 2018; Roudier et al., 2014). For instance, a community might work together to test different crop varieties under local soil and climatic conditions to identify those that perform best, thereby creating a locally adapted solution to agricultural challenges posed by climate change. Interventions may also incorporate an overt emphasis on addressing the gender inequalities in access to knowledge and control over resources between men and women in agriculture or related activities (Gumucio et al., 2020).

**Figure 1:**
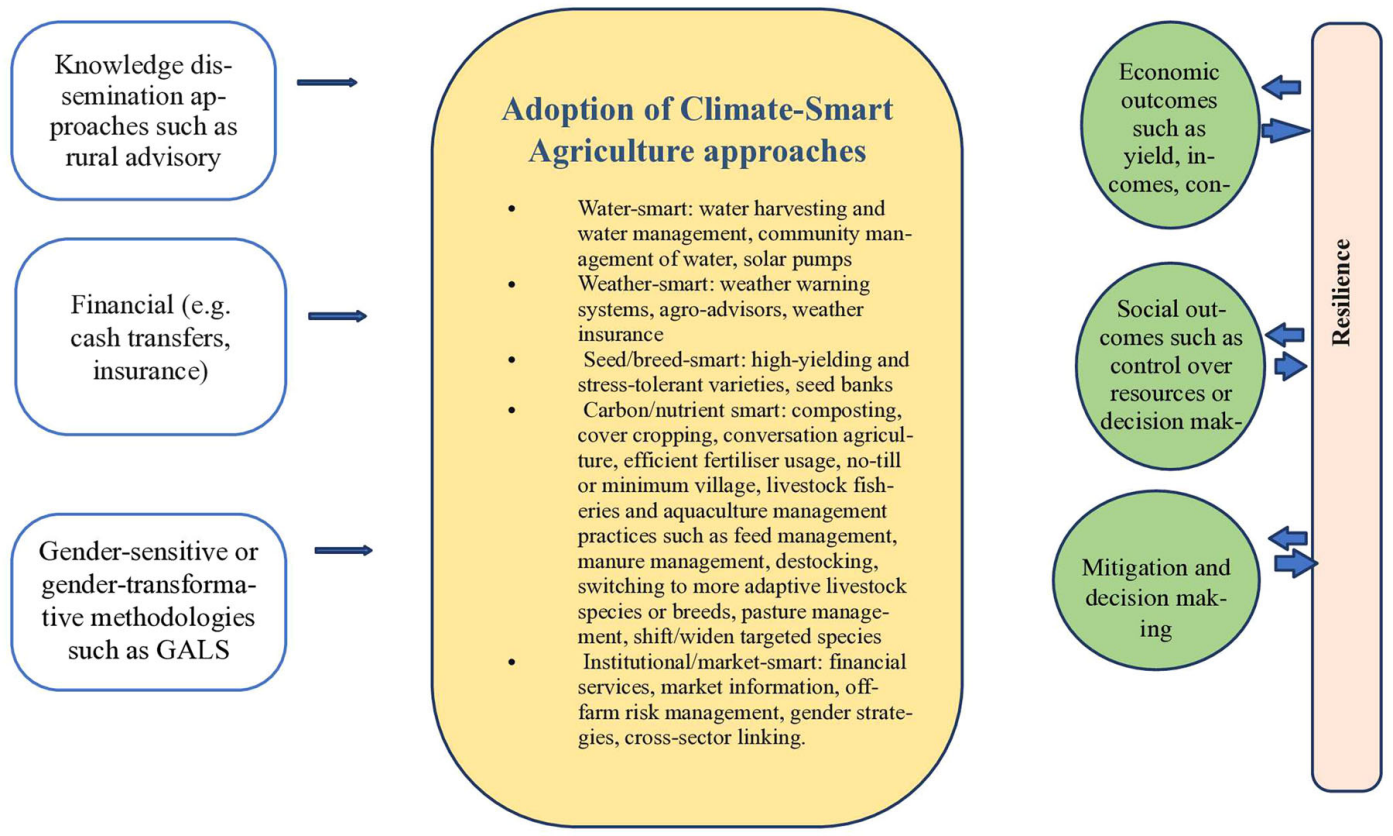
Logic model. *Source*: Lopez‐Avila et al. (2017) and World Bank, IFAD and FAO (2008).

The causal pathway through which CSA promotion interventions for land, soil, water, and biodiversity management can enhance women's resilience is shown in Figure 1. Resilience refers to “the ability to persist in and survive a disaster with minimal influence and destruction. It includes the ability to lessen or evade damages, encompass the impacts of hazards, and bounce back with slight disturbances” (Cutter et al., 2008). Adaptive capacity shows the ability of a household to adapt and cope with a hazard, in this case, climate‐induced shocks and stresses, such as drought, floods, and climate variability. This enables families to continue performing their essential functions during such hazards (FAO, 2014).

The first step toward encouraging farmer communities to adopt CSA technologies is building awareness about climate change risks and uncertainties and how these risks can be countered by the preparedness of systems built on CSA practices. Knowledge dissemination approaches, such as FFS, may thus provide information and awareness regarding appropriate CSA approaches (IRRI and CRISP, 2020). Depending on a community's level of engagement, local solutions may also be incorporated into its suite of CSA approaches for land/soil, water, and biodiversity management. For example, the financial bottlenecks in adopting CSA approaches can be handled by providing microfinance assistance or subsidies on inputs, such as seeds, fertilizers, and equipment. Crop insurance is increasingly used to help farmers manage climate risk. Institutional arrangements, such as land titling and community infrastructure projects (e.g., irrigation), may also help overcome barriers to adoption (Makate, 2019).

The above‐mentioned interventions may aid in the adoption of context‐appropriate CSA approaches, which can increase the knowledge score and understanding of the benefits of CSA approaches, subsequent adoption, and long‐term sustainable economic, social, and environmental outcomes. These economic outcomes may include increased yields, incomes, and consumption/food security.

### 3.4 Why it is important to do this review

CSA has emerged as a holistic approach that aims to increase productivity, enhance resilience, and reduce GHG emissions in agricultural systems. CSA practices not only mitigate climate change but also contribute to sustainable development goals (SDGs) (IPCC, 2019; World Bank, FAO and IFAD, 2015). The SDGs of climate action (SDG 13), responsible consumption and production (SDG 12), zero hunger (SDG 2), and poverty eradication (SDG 1) are closely linked to climate‐smart approaches to agriculture, land, forestry, and water management (IPCC, 2021). These SDGs depend on sustainable agricultural practices, such as minimizing water waste and using organic farming techniques, which can help reduce GHGs released into the atmosphere. Sustainable land management practices, such as reforestation and better land management, can reduce soil erosion and plant more carbon‐absorbing trees. A crucial part of building a CSA evidence base is identifying and synthesizing the existing evidence base on the effectiveness of interventions promoting CSA approaches and identifying the key vulnerabilities. A global evidence map of climate change adaptation initiatives, including in agriculture, has also been published (Berrang‐Ford et al., 2021). Systematic reviews have been conducted or planned on the effects of CSA technologies (Rosenstock et al., 2016; Bui & Vu, 2020). Researchers have also reviewed interventions that promote smallholders' adoption of agricultural technology using top‐down and bottom‐up approaches (Korth et al., 2014; Waddington et al., 2014). In addition, systematic reviews have incorporated evidence of the effects of changing institutional arrangements, such as contract farming (Ton et al., 2018), and explicitly examined the impacts on women farmers, including farmer field schools (Waddington et al., 2014; van den Berg et al., 2015) and land reform (Lawry et al., 2017). Another review examined interventions designed to empower communities in the NRM (Waddington et al., 2019). A previous systematic review examined the impact of aquaculture interventions on women's productivity, income, nutrition, and empowerment (Gonzalez et al., 2021). A systematic map summarizing published evidence of the gender composition of forestry and fishery management groups (Leisher et al., 2016) and an evidence gap map of agricultural innovation (Lopez‐Avila et al., 2017). However, we did not find existing or planned systematic reviews focusing on the effect of interventions promoting CSA practices on smallholders' and women farmers' knowledge and subsequent adoption of CSA practices.

This review will examine various interventions, such as capacity‐building programs, financial incentives, and extension services, to evaluate their impact on knowledge acquisition and adoption rates of CSA approaches by smallholder farmers in LMICs.

## 4 OBJECTIVES

The primary objective of this review is to synthesize evidence of the effectiveness of interventions promoting CSA to enhance smallholder farmers' knowledge of the benefits of CSA approaches, subsequent adoption of CSA, and disadoption of harmful agricultural practices in LMICs.

We also aimed to identify whether there are any differences in the impact of these interventions on women and men farmers (where and if studies reported an impact for women and men farmers separately) (O'Neill et al., 2014).

## 5 METHODS

### 5.1 Criteria for considering studies for this review

#### 5.1.1 Types of studies

Eligible studies include those in which the authors used a control or comparison group (Littell, 2018):

- Randomized or Quasi‐Random Assignment: Eligible studies should have participants randomly or quasi‐randomly assigned to different intervention groups.
- Non‐Random Assignment with Matching or Statistical Control: If random assignment is not feasible, studies can still be eligible if participants are assigned non‐randomly but matched by relevant characteristics. Alternatively, statistical methods (such as propensity score matching) can be used to control for differences between groups.
- Before‐After Studies: Controlled before‐after studies (with a pre‐intervention and post‐intervention assessment) are eligible.
- Combinations of Approaches: Eligible studies may use combinations of the above approaches. Examples include pair‐matched randomization, randomized encouragement using instrumental variables, fuzzy regression discontinuity using instrumental variables, or propensity score‐weighted difference‐in‐differences.

##### Examples of studies included and excluded

Supporting Information: Appendix 1 lists the studies in the reviews, and Supporting Information: Appendix 2 lists the excluded studies.

#### 5.1.2 Types of participants

Eligible participants were women and men farmers engaged in agriculture and natural resource management in LMICs, as defined by the World Bank categorization at the time the intervention was conducted.

#### 5.1.3 Types of interventions

Eligible interventions are those that promote existing, new, or improved climate‐smart agricultural approaches.

- Knowledge dissemination and capacity‐building approaches such as Farmer's Field Schools or their modification, social networking and peer learning, information and communication technologies, group and individual training and demonstration, and agriculture extension services.
- Financial approaches, including credit and subsidies such as cash transfers, vouchers, matching grants, and crop insurance.
- Institutional arrangements such as collectivization (e.g. farmer cooperatives and federations).

*Comparators* included business‐as‐usual access to conventional agricultural services, including no access or promotion of non‐climate smart approaches, different interventions promoting CSA, or interventions with different intensities.

#### 5.1.4 Types of outcome measures

This review included three key outcomes related to CSA: knowledge and awareness about appropriate CSA approaches, adoption of proper CSA practices, and disadoption of harmful practices, agriculture outcomes (e.g., yield), and social outcomes (e.g., time use).

#### 5.1.5 Duration of follow‐up

Any follow‐up duration was eligible, and multiple outcomes for multiple follow‐ups were coded to be used as “synthetic effects” or synthesizing effects by time period where sufficient studies report multiple similar follow‐ups.

### 5.2 Search methods for identification of studies

The electronic searches of the selected databases were accompanied by a gray literature search using organizational websites. Systematic review databases were also searched. Hand searches of selected journals and articles were also conducted.

#### 5.2.1 Electronic searches

The electronic searches of selected databases were accompanied by gray literature searches using organizational websites. Only studies published in English were selected for the review.

*Electronic searches*: The search string was designed according to our research questions and used in a series of databases known to cover agricultural literature. The following databases were searched: AgEcon, CAB Abstracts, Web of Knowledge (Social Sciences Citation Index [SSCI] and Social Science Conference Proceedings), SCOPUS, International Bibliography of the Social Sciences, AGRIS, EconLit, US National Agricultural Library (Agricola) and EBSCO multifile groups of databases: Academic Search Research and Development, Africa‐Wide Information, SocIndex. Supporting Information: Appendix 1 provides the search string for the Web of Science Core Collection (SSCI).

*Searching other resources*: International Initiative of Impact Evaluation (3IE), Epistemonikos, DFID Research for Development (R4D), IMMANA grant database, 3ie impact evaluation repository and The World Bank IEG evaluations, OECD/DAC Evaluation database, Google Scholar, OpenGrey, Networked Digital Library of Theses and Dissertations (NDLTD), were also searched www.theses.org.

We also searched the organizational websites and repositories of the CGIAR group, IFAD, IIED, FAO, AgriProFocus, BMGF, Donor Committee for Enterprise Development, Swiss Agency for Development and Cooperation, Department for International Development (DFID), IPA and J‐PAL, and the USAID Development Experience Clearinghouse.

**Figure 2:**
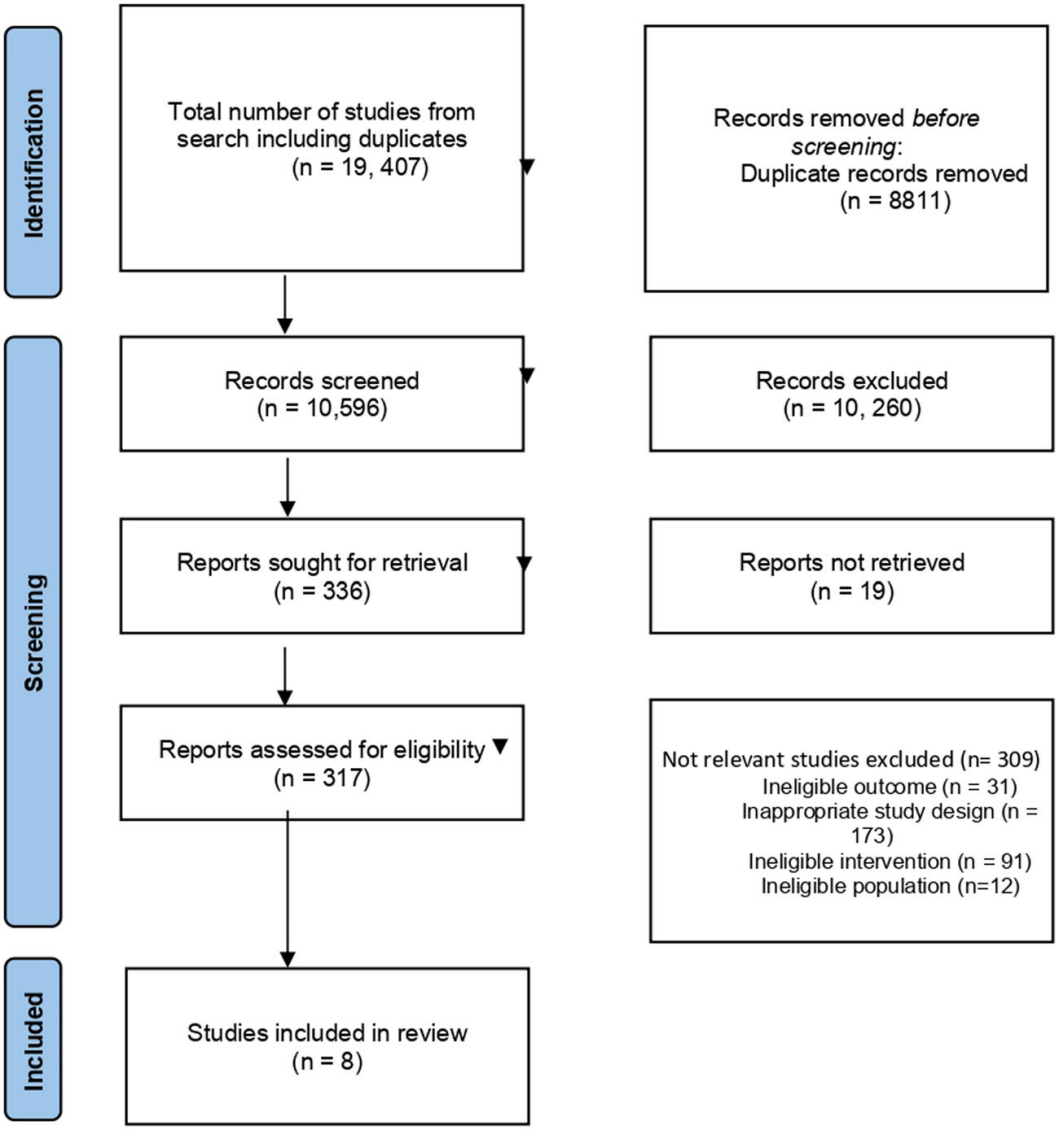
Prisma flow chart of search process.

Conference proceedings and papers from the Agriculture, Nutrition and Health Academy Conference, the Center for the Study of African Economies (CSAE) Conference, the North East Universities Development Consortium (NEUDC) Conference and the World Bank Economic Review were also searched to identify eligible conference papers. Citation searches in Web of Science and Google Scholar for included studies were conducted. Finally, we examined the reference lists in relevant global maps (Lopez‐Avila et al., 2017; Berrang‐Ford et al., 2021). Figure 2 shows the PRISMA flowchart of study selection.

### 5.3 Data collection and analysis

#### 5.3.1 Selection of studies

Eppi‐Reviewer software was used for data management and analysis. All identified studies were imported to the Eppi‐Reviewer for screening, followed by data extraction. Two researchers independently screened The identified studies at the title & abstract and full‐text stage using a pre‐validated screening tool (Supporting Information: Appendix 2). The screening tool was piloted to screen for 100 studies. The disagreements at both screening stages were resolved by discussion and, if necessary, arbitrated by a third reviewer.

#### 5.3.2 Data extraction and management

We extracted a range of data, including bibliographic details, outcomes, interventions, period covered and study design using a pre‐validated data extraction tool (Supporting Information: Appendix 3). Two researchers independently extracted the data, and the data extraction reports were matched for agreements.

##### Assessment of risk of bias in included studies

For randomized and non‐randomized studies, the potential risk of bias in the included studies was assessed using a tool adapted from Waddington et al. (2012) and Stewart et al. (2014), which articulates bias domains around confounding, selection bias, departures from intended interventions, and bias in measurement, and reporting bias (Supporting Information: Appendix 4).

Risk of bias ratings were assigned for each of the seven domains, varying from low to moderate to high.

##### Measures of treatment effect

Effect size estimates with 95% confidence intervals (95% CIs) were extracted from included studies. Effect sizes were measured as mean differences (where studies use the identical continuous outcome measured in the same units), standardized mean differences (SMDs), or, in the case of dichotomous outcome variables, odds ratios, with their standard errors and 95% CIs (Waddington & Cairncross, 2021; Cumpston et al., 2019).

##### Assessment of heterogeneity

We assessed heterogeneity among the included studies by calculating the *I* ^2^ statistic (Egger et al., 1997), which quantifies the percentage of total variation across studies that is due to heterogeneity rather than chance (Langan et al., 2019).

##### Data synthesis

Effect sizes were pooled statistically using inverse‐variance weighted random‐effects meta‐analysis, using the mean and meta‐reg commands in Stata Version 16. Using forest plots, we presented effect sizes and 95% CIs. Where constructs were considered sufficiently similar, we estimated pooled effect sizes across studies using inverse‐variance weighted random effects meta‐analysis using R software (R). We undertook a sub‐group analysis by sex.

##### Treatment of qualitative research

We did not include qualitative studies in our review.

## 6 RESULTS

### 6.1 Description of studies

#### 6.1.1 Results of the search

The search initially identified 19,407 studies. From these initial search results, 8811 duplicates were removed, leaving 10,596 records. Supporting Information: Appendix 2 provides initial hits for each database search. After screening the title and abstracts according to inclusion criteria, a further 10,260 records were removed, leaving 336 records. The remaining 336 records were screened for the full text. After double‐screening all full‐text documents, eight papers were included. The search results are summarized in Figure 2.

#### 6.1.2 Included studies

This section summarizes the eight included studies and their characteristics, including region, CSA practices, target group of interventions, and outcomes. Note that the studies will be referred to by the first author's last name and year of publication.

*Region*: Sub‐Saharan Africa was represented by three studies: Osumba et al. (2021) covered Kenya, Tanzania, and Uganda; Djido et al. (2021) from Ghana; Nyasimi et al. (2017) included Tanzania. Four studies were from South Asia, specifically Bangladesh (Chowdhury et al., 2015) and India (Dar et al., 2020; Mancini, 2006; Sharma et al., 2019). East Asia and the Pacific were represented by China (Guo et al., 2015). Neither Europe, Central Asia, the Middle East, nor North Africa were represented in the studies.

##### CSA practices characteristics

A wide ranhe of CSA practices were promoted in the studies, including integrated pest management (IPM), the use of high‐yield, climate‐resilient crop varieties, soil fertility management, water management, and mitigation of climate change effects. Dar et al. (2020) aimed to promote knowledge about high‐yield, climate‐resilient crop varieties, especially their benefits, to smallholder farmers. Researchers evaluated the impact of quality seed production training on male and female farmers' awareness and adoption of climate‐resilient or stress‐tolerant rice varieties (STRVs) in abiotic‐stress‐prone states in India most affected by droughts. A study conducted by Djido et al. (2021) examined the relationship between weather and climate information services (weather forecasts, call centers, agro‐advisories, and input and output market prices) in Ghanaian smallholder farmers' adoption of five CSA practices (eradication of erosion, pest management, pest‐resistant crops, water management, and multiple cropping). Nyasimi et al. (2017) evaluated the effectiveness of the “farms of the future” (FOTF) approach developed by the Consultative Group for International Agricultural Research Program on Climate Change, Agriculture, and Food Security. Through farm visits and exchanges between spatial analogs, this approach is based on peer learning (farmer‐to‐farmer interaction) and connects farmers to their possible climate futures. The CSA practice implemented in the study by Chowdhury et al. (2015) involves the utilization of video‐mediated learning to enhance farmers' understanding of botanical pesticide innovation. Video‐mediated learning refers to the use of instructional videos as a means of disseminating knowledge and providing training to farmers. This practice is considered climate‐smart as it promotes sustainable agriculture by reducing the use of chemical pesticides, minimizing environmental impact, and increasing the resilience of farming systems to climate change. The climate‐smart practice highlighted in the study by Mancini (2006) involves the adoption of IPM techniques through FFS. IPM is an environmentally friendly approach to pest management that integrates various strategies, including biological control, cultural practices, and judicious use of pesticides. It emphasizes the reduction of chemical inputs, promotes biodiversity conservation, and enhances the overall sustainability of farming systems.

Guo et al. (2015) evaluated the effectiveness of the FFS approach in enhancing farmer knowledge acquisition, changes in agricultural practices, crop yields, and the adoption of integrated low‐carbon farm management that is still yield enhancing. The study by Sharma et al. (2019) focused on utilizing a social marketing approach to change water use behavior among rural communities in Punjab, India. The study highlights the significance of promoting sustainable water management practices and the role of social marketing in shaping behavior towards water conservation.

##### Intervention characteristics

The included studies examined three of the six intervention types in Table 1. All studies reported knowledge dissemination and capacity‐building interventions, of which four involved farmer field schools or their modifications as a component of an intervention (Guo et al., 2015; Mancini, 2006; Nyasimi et al., 2017; Osumba et al., 2021).

**Table 1:** Intervention characteristics of selected studies.

Type of response to climate change	Examples of specific activities associated with each response to climate change	Type of promotional interventions	Studies reporting responses to climate change
Climate‐resilient crop seeds	Stress tolerant rice variety (STRV)	Quality seed production training (QSP)	Dar et al. (2020)
Use of fertilizers and pesticides	Agronomic practices	Weather and climate information services (weather forecasts, call centers, agro‐advisories, input and output market prices) Farmer field schools Video‐mediated learning	Djido et al. (2021) Guo et al. (2015); Mancini et al. (2006) Chowdhury et al. (2015)
Modified planting activities	Multiple cropping practices	Weather and climate information services (weather forecasts, call centers, agro‐advisories, input and output market prices)	Djido et al. (2021); Nyasimi (2007)
Irrigation and water management	Water conservation strategies, irrigation, micro irrigation, water harvesting and improving drainage	Weather and climate information services (weather forecasts, input and output market prices)	Djido et al. (2021); Sharma et al. (2019); Osumba et al. (2021)

The FFS intervention in Guo et al. (2015) was designed to address rice farmers' specific challenges in China. The training curriculum was developed based on the best available scientific knowledge and local agricultural practices. It covered a wide range of topics related to rice production, including land preparation, seed selection, planting techniques, water management, pest and disease control, nutrient management, and harvesting methods. A team of agricultural experts, extension workers, and trained facilitators played a crucial role in implementing the FFS intervention. They facilitated the training sessions, organized field visits, and provided technical guidance and support to the participating farmers. The training sessions were conducted participatory, encouraging active engagement and knowledge sharing among the farmers. The FFS intervention also incorporated hands‐on learning activities. The intervention in the study by Mancini (2006) involved the establishment of IPM FFS within the cotton‐growing regions of Southern India. These schools served as a platform to disseminate knowledge and practical training on IPM techniques to cotton growers. The intervention aimed to address the challenges faced by farmers, such as pesticide overuse, environmental degradation, and health hazards, by promoting sustainable pest management practices. Osumba et al. (2021) focused on establishing FFS that adopted innovative and climate‐resilient agribusiness practices. The FFS sessions were facilitated by trained agricultural extension officers who acted as mentors and facilitators. They provided guidance on climate‐resilient farming techniques, such as improved irrigation methods, crop diversification, soil conservation practices, and the use of weather information for decision‐making. The Farms of the Future (FotF) approach was used in the study by Nyasimi et al. (2017), which is a participatory and farmer‐centric methodology that focuses on knowledge‐sharing, capacity building, and technology transfer. The intervention involved a series of activities designed to engage farmers, extension workers, and local stakeholders in the process of adopting CSA technologies and practices.

Sharma et al. (2019) covered the use of participatory action research by implementing a social marketing campaign focused on raising awareness, changing attitudes, and promoting behavior change related to water use in the rural village of Punjab. The campaign consisted of various components to engage the target population and encourage them to adopt sustainable water practices. Two studies included video‐mediated training and demonstration for groups and individuals about botanical pesticide innovation and use (Chowdhury et al., 2015; Dar et al., 2020). In the study, Chowdhury et al. (2015) focused on utilizing video‐mediated learning to disseminate knowledge and skills related to botanical pesticide innovation among farmers in Bangladesh. The study by Dar et al. (2020) was the only study aimed at intervention incorporating a gender‐focused approach, recognizing the unique challenges and opportunities faced by women in agriculture. It seeks to promote gender equality by ensuring equal participation and representation of women in the training sessions. Additionally, the intervention addresses specific gender‐related constraints and provides tailored solutions to support women farmers in adopting climate‐resilient seeds. The study, Djido et al. (2021) focus on examining the extent to which weather and climate information services contribute to the adoption of CSA practices in Ghana. The interventions involved the provision of weather and climate information services to a selected group of farmers in Ghana. These services included timely weather forecasts, climate predictions, and advisory messages tailored to the specific needs of the farmers. The information was delivered through various channels, including radio, mobile phones, and community meetings.

##### Outcome characteristics

Studies included in this review examined seven outcomes (Table 2); however, the number of studies reporting the outcome categories is limited, with only one study for each of management (Guo et al., 2015), time use (Mancini, 2006), and yield (Guo et al., 2015; Mancini, 2006). Among the four studies that evaluated the knowledge gains, three of them reported changes in farmers' knowledge scores about IPM (Chowdhury et al., 2015; Guo et al., 2015; Mancini, 2006) and one investigated farmers' knowledge and adoption of climate‐resilient rice varieties (STRVs) or climate‐resilient rice varieties (Dar et al., 2020). The adoption of IPM and botanical pesticides was investigated in three studies (Chowdhury et al., 2015; Guo et al., 2015; Mancini, 2006). An evaluation of the effectiveness of video‐mediated learning sessions in changing the attitudes of smallholder farmers about the use of botanical pesticides was conducted by Choudhury et al. (2015). Two studies (Chowdhury et al., 2015; Mancini, 2006) reported the disadoption of harmful practices, such as pesticide overuse. The impact of the adoption of CSA practices on time use was examined in one study (Mancini, 2006), and it found that such adoption did not result in an increase in the total amount of labor time in the economy but rather required women to be the ones to do most of the work. The effects on decision‐making and yield were explained in only one study (Mancini, 2006).

**Table 2:** Outcome type and sub‐types included in meta‐analysis.

Outcome type	Examples of outcome sub‐types included	Studies reporting outcome
Knowledge	Knowledge score (pest management), knowledge of helpful practices (pesticide overuse), knowledge of harm (chemical pesticides), and farmers' knowledge and adoption of climate‐resilient rice varieties or STRVs	Chowdhury et al. (2015), Dar et al. (2020), Guo et al. (2015), Mancini (2006)
Attitudes	Agreement with beneficial practices	Chowdhury et al. (2015)
Disadoption (harmful practice)	Number of sprays during first 40 days and use of pesticide	Chowdhury et al. (2015); Mancini (2006)
Adoption (favored practice)	Adoption of CSA, use of botanical inputs and safe storage of inputs	Chowdhury et al. (2015); Dar et al. (2020); Mancini (2006)
Management	Sustainable farm	Guo et al. (2015)
Time use	Total labor use, labor use for specific tasks (e.g. pesticide, IPM tasks) and the female share of total work	Mancini (2006)
Yield	Kilograms per hectare	Mancini (2006)

##### Subgroup analysis

Four studies provided information about gender differences between the outcomes. Figures 4 and 5 plot the group differences in the effect on the knowledge and adoption of CSA practices. The effects varied between males and females. Males performed better in two out of three studies, however, the pooled effect was insignificant. As for adoption practices, the impact of training was consistently higher for females across all four studies.

#### 6.1.3 Excluded studies

The exclusions are recorded in the PRISMA diagram above. The excluded studies, along with their reasons for exclusion, are presented in Annex A. Common exclusion reasons were that the study was not an impact evaluation, presented a protocol without associated results, did not focus on women farmers or did not have gender‐disaggregated data, did not consider CSA promotion interventions, or used only qualitative data.

### 6.2 Risk of bias in included studies

**Figure 3:**
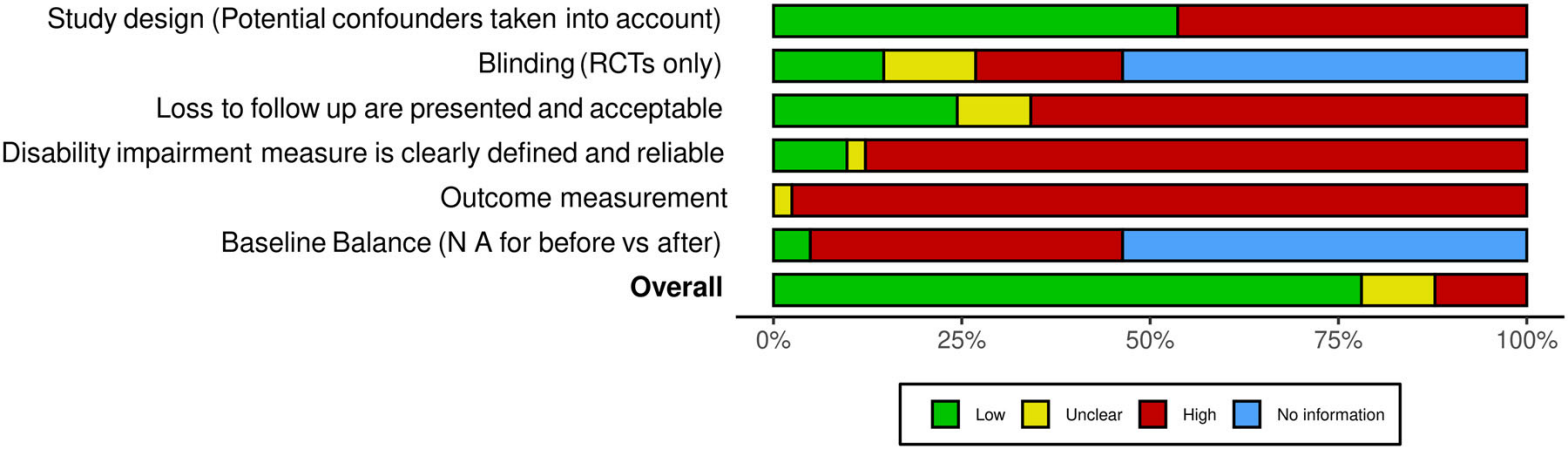
Over all risk of bias.

We identified eight impact evaluations assessing the effects of implementing CSA promotion interventions to enhance women farmers' agricultural outcomes and resilience in LMICs. Figure 3 presents a summary of the evaluation results of the risk of bias of the included studies.

Overall, the samples of the included studies had a high risk of bias. Only two studies had a low risk of bias (Dar et al., 2020; Guo et al., 2015). Most studies had a high risk of bias (*n* = 5), and one had a moderate risk of bias (Mancini, 2006). Selection bias and reporting bias due to incomplete outcome reporting were the main reasons for this body of evidence's high overall risk of bias. The two randomized controlled trials (RCTs) (Guo et al., 2015; Dar et al., 2020) reported attrition rates of over 20%, indicating a high risk of attrition bias.

Only one study (Djido et al., 2021) attempted to rigorously address selection bias using endogenous switching regression to account for endogeneity bias and the effects of unobservable covariates. All included studies were analyzed based on the farm or household survey data consistently applied across their treatment and control groups. The risk of performance bias between the groups was small.

Most of the studies did not explicitly discuss potential spillover effects. Furthermore, most studies selected the treatment and control groups from the same population, not geographically separated. Some studies had a high risk of bias due to issues about self‐selection into the program and the analysis is based only on cross‐sectional data.

Another shortcoming is that the evidence base included only two RCTs, an essential method for addressing this potential source of bias.

### 6.3 Synthesis of results

The meta‐analysis results focus on three key outcomes related to CSA: knowledge and awareness about appropriate CSA approaches, adoption of proper CSA practices, and disadoption of harmful practices. The effects on agricultural productivity in the form of yields and time spent on specific tasks were also examined. All pooled effects were calculated using random‐effects models, as the evidence was related to different populations at various locations and times. We also conducted subgroup analysis by gender. Table 3 shows these categories, which we combined to perform the meta‐analysis using independent estimates. The differences between effect sizes across outcome types were measured using the *I*² statistic and the corresponding *p*‐value.

**Table 3:** Summary of findings table.

Outcome	Effect	Summary
Knowledge	*d* = 0.30 (0.13 to 0.46) *n* = 4 *I*² = 75% Egger's test −3.47 (*t* = −2.15, *p* = 0.16)	Moderate effect based on a small number of studies with moderate heterogeneity and small publication bias
Disadoption	*d* = 0.21 (0.08 to 0.50) *n* = 2 *I*² = 80% Eggers test Too small sample size	Small effect based on a small number of studies with high heterogeneity and potential publication bias
Adoption	*d* = 0.25 (0.03 to 0.48) *n* = 3 *I*² = 81% Eggers test Too small sample size	Small effect based on a small number of studies with high heterogeneity and potential publication bias

In the following figures, forest plots are presented for these outcomes. We examined the overall effects of the interventions on the desired outcome types.

### 6.4 Meta‐analysis

#### 6.4.1 Knowledge

**Figure 4:**
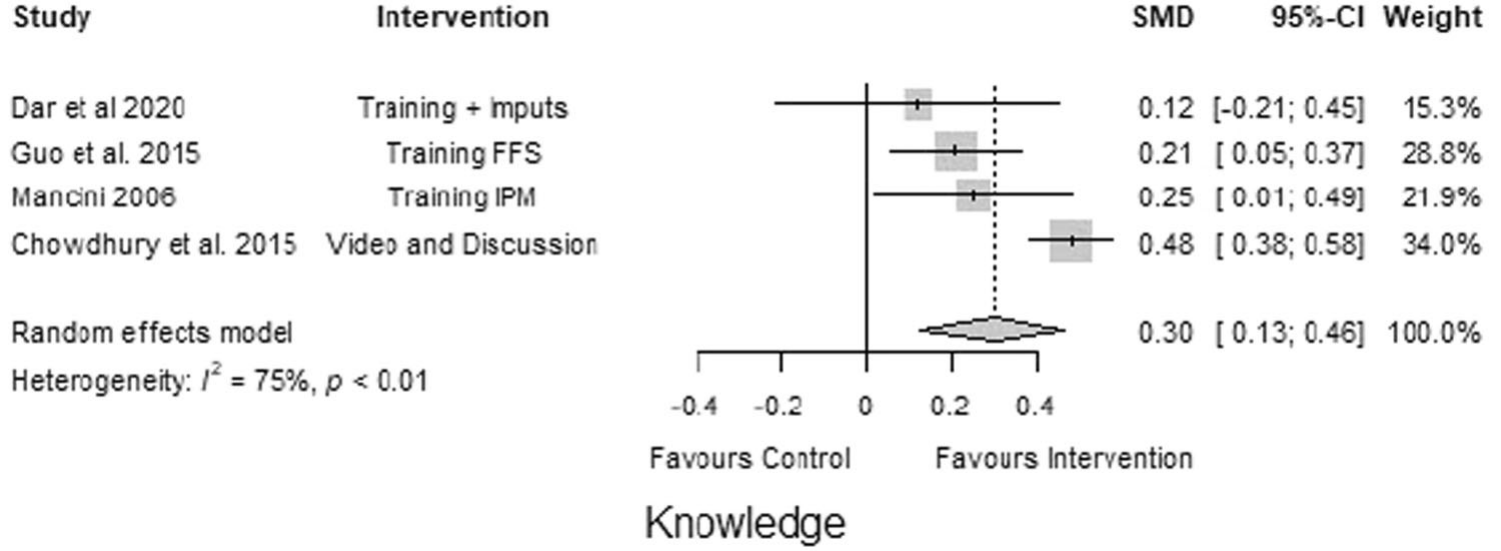
Forest plot of studies reporting knowledge outcomes.

Four studies (Chowdhury et al., 2015; Dar et al., 2020; Guo et al., 2015; Mancini, 2006) explained the effects of knowledge dissemination approaches, such as agricultural extension services and video‐mediated farmer‐to‐farmer learning, on the knowledge of smallholder farmers, which were measured using the changes in their knowledge scores. The effect sizes for the knowledge outcomes are expressed as SMDs; the knowledge change of farmers receiving knowledge dissemination and capacity‐building interventions was compared with that of farmers in the non‐intervention comparison group. This is presented here as the changes in the standard deviations of the outcomes. All four studies showed a positive effect of the interventions on farmers' knowledge change about helpful CSA practices. The interventions had a moderate positive effect on farmers' knowledge of CSA practices (SMD = 0.30, CI = 0.14 to 0.46, *p* < 0.01), but the effect varied significantly between studies (*I*² = 75%) (Figure 4). This difference could be attributed to the different methods used in each study, such as the intensity of training, the type of information provided, and the frequency of follow‐up.

#### 6.4.2 Adoption and disadoption

**Figure 5:**
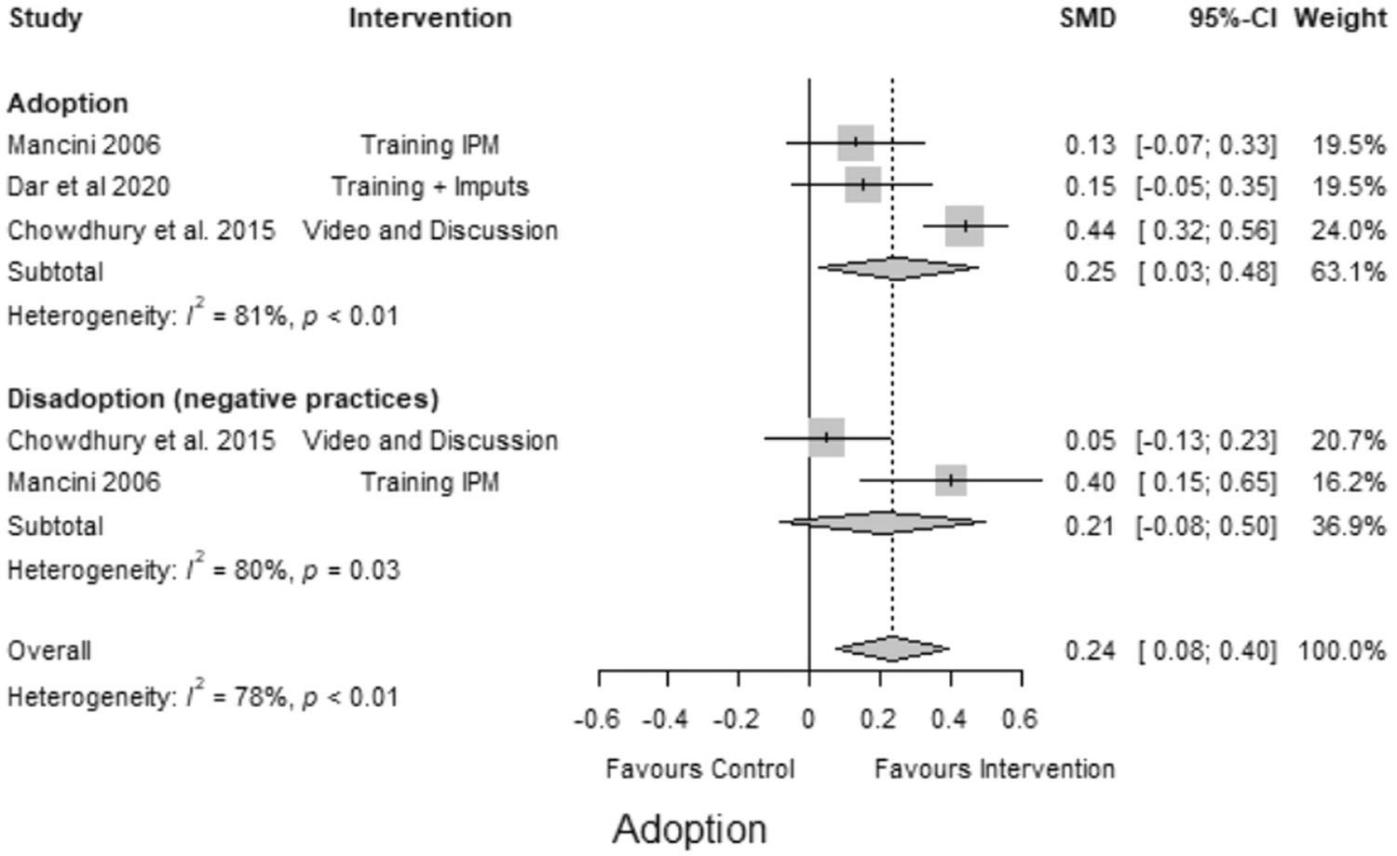
Forest plot of studies reporting adoption of climate‐smart agriculture (CSA) practices.

We examined the impact of interventions that promote the adoption of CSA practices, such as using climate‐resilient seeds and reducing harmful practices (e.g., pesticide use and number of sprays) (Figure 5). Chowdhury et al. (2015), Dar et al. (2020), Mancini (2006) investigated the adoption of these good practices. The effects reported by the two other studies were small and insignificant despite being positive, and only one study (Chowdhury et al., 2015) reported a statistically significant improvement. The interventions had a small positive effect on farmers' adoption of CSA practices and indicated heterogeneity. This indicates that the reported improvement was inconsistent across all studies and that different factors may have contributed to the variance in the effects. These factors may include differences in the sample size, the type of industry, or the level of implementation (SMD = 0.25, CI = 0.03 to 0.48, *k* = 3, *I*² = 81%, *p* < 0.001) (Figure 5).

The results showed that farmers who received targeted interventions were more likely to adopt CSA practices than those who did not. This indicates that targeted interventions are an effective way to promote CSA among farmers.

Mancini (2006) was the only study with a significant positive effect on reducing harmful practices. However, the pooled effect size was small and insignificant (SMD = 0.21, CI = 0.08 to 0.50, *k* = 2, *I* ^2^ = 80, *p*: 0.03).

#### 6.4.3 Time use

**Figure 6:**
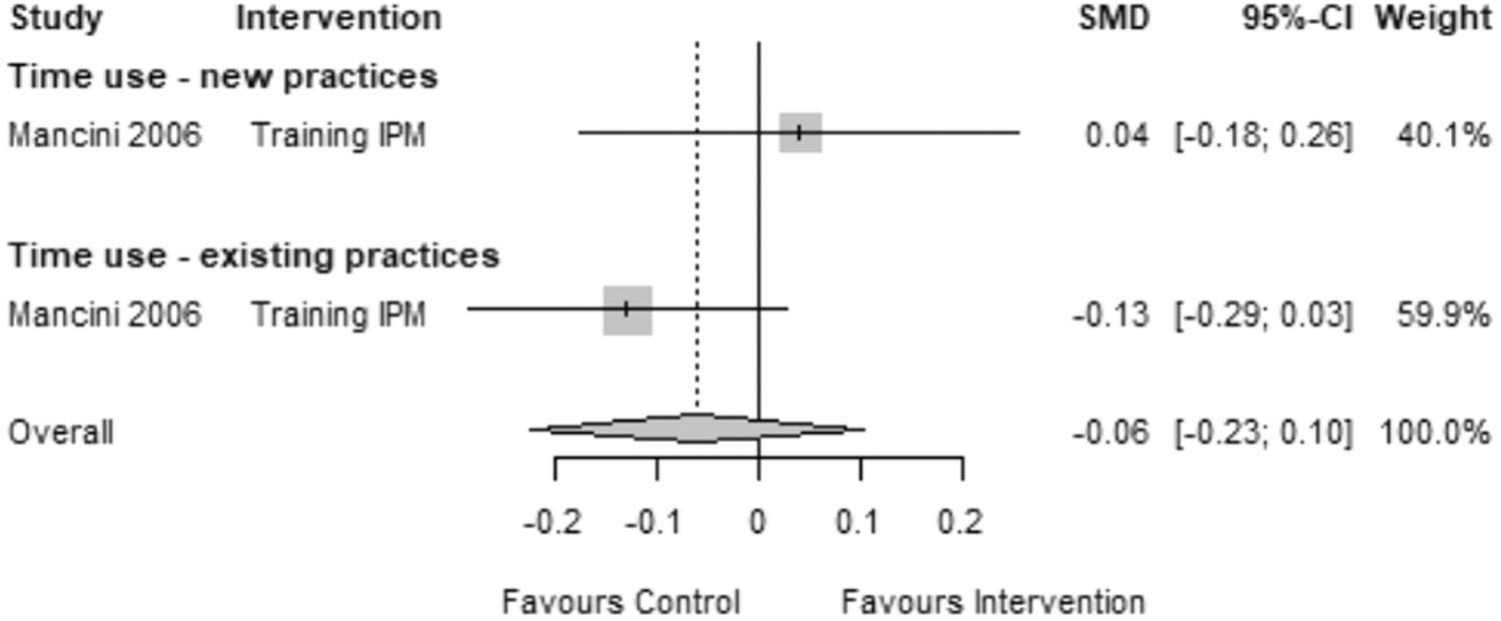
Forest plot of studies reporting time use.

In only one study (Mancini, 2006), the labor time burden was measured; the author estimated increases in labor time due to IPM adoption. Compared with farms using pesticides (SMD = 0.04, CI = 0.18 to 0.26), IPM cotton farms experienced increased labor time (SMD = 0.13, CI = 0.29 to 0.03) after the intervention (Figure 6). Although there was a difference, it was not statistically significant. Women's availability could influence the adoption of IPM since the time burden descended upon women's household members.

#### 6.4.4 Attitude

**Figure 7:**
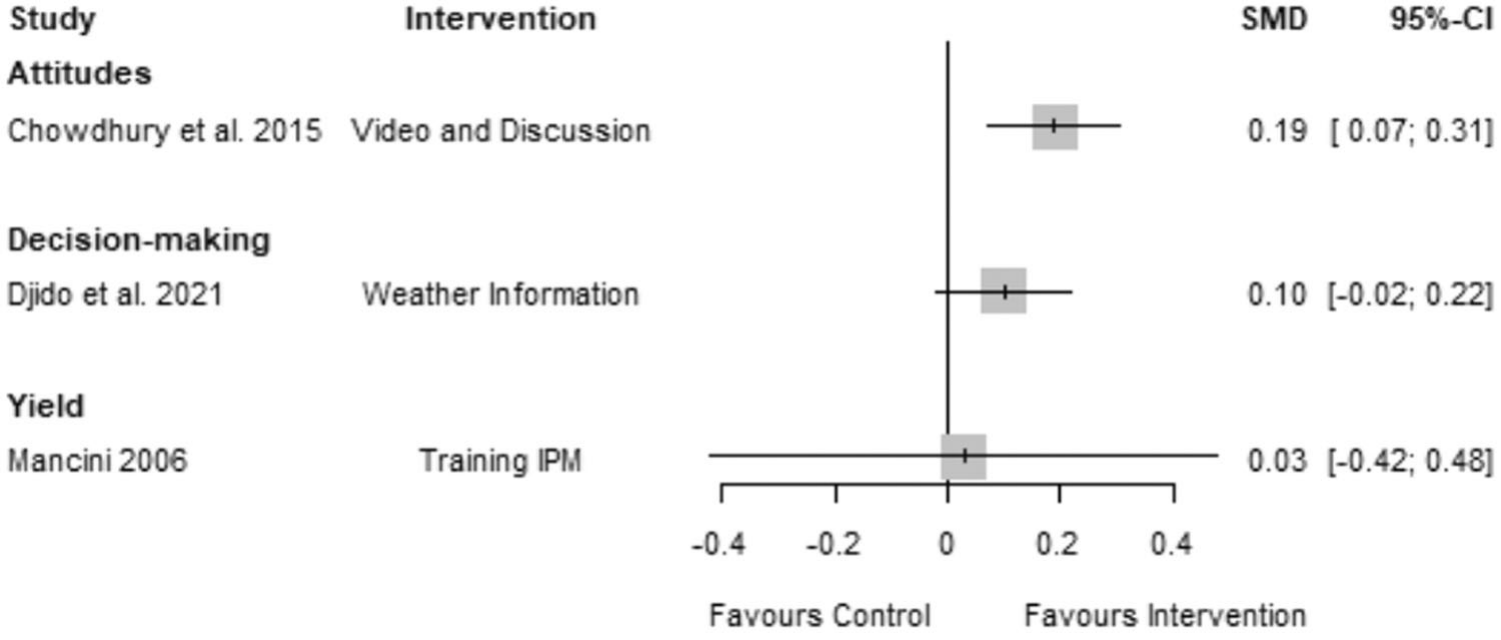
Forest plot reporting studies on attitude, yield and decision‐making.

The forest plot indicates that the intervention positively affected farmers' attitudes (SMD = 0.21, CI = 0.07 to 0.35) (Figure 7). The forest plot shows that the difference between the mean attitude of farmers in the experimental and control groups was statistically significant. The effects on decision‐making and yield were insignificant.

*Overall*, the interventions improved the farmers' understanding of the CSA principles and practices, changed their attitude towards CSA and helped them adopt the practices. This is evidenced by the statistically significant results seen in Table 3. There was an average effect size of 0.30 for training on knowledge. Comparatively, the effects of adoption practices and attitude were much lower on average (0.24 and 0.19, respectively), although they were still positive and significant on average. Despite the above consensus on the direction of the effects, the studies presented considerable heterogeneity in effect size. This indicates that although the interventions had an overall positive effect, the results were inconsistent across all studies.

#### 6.4.5 Subgroup analysis by gender

**Figure 8:**
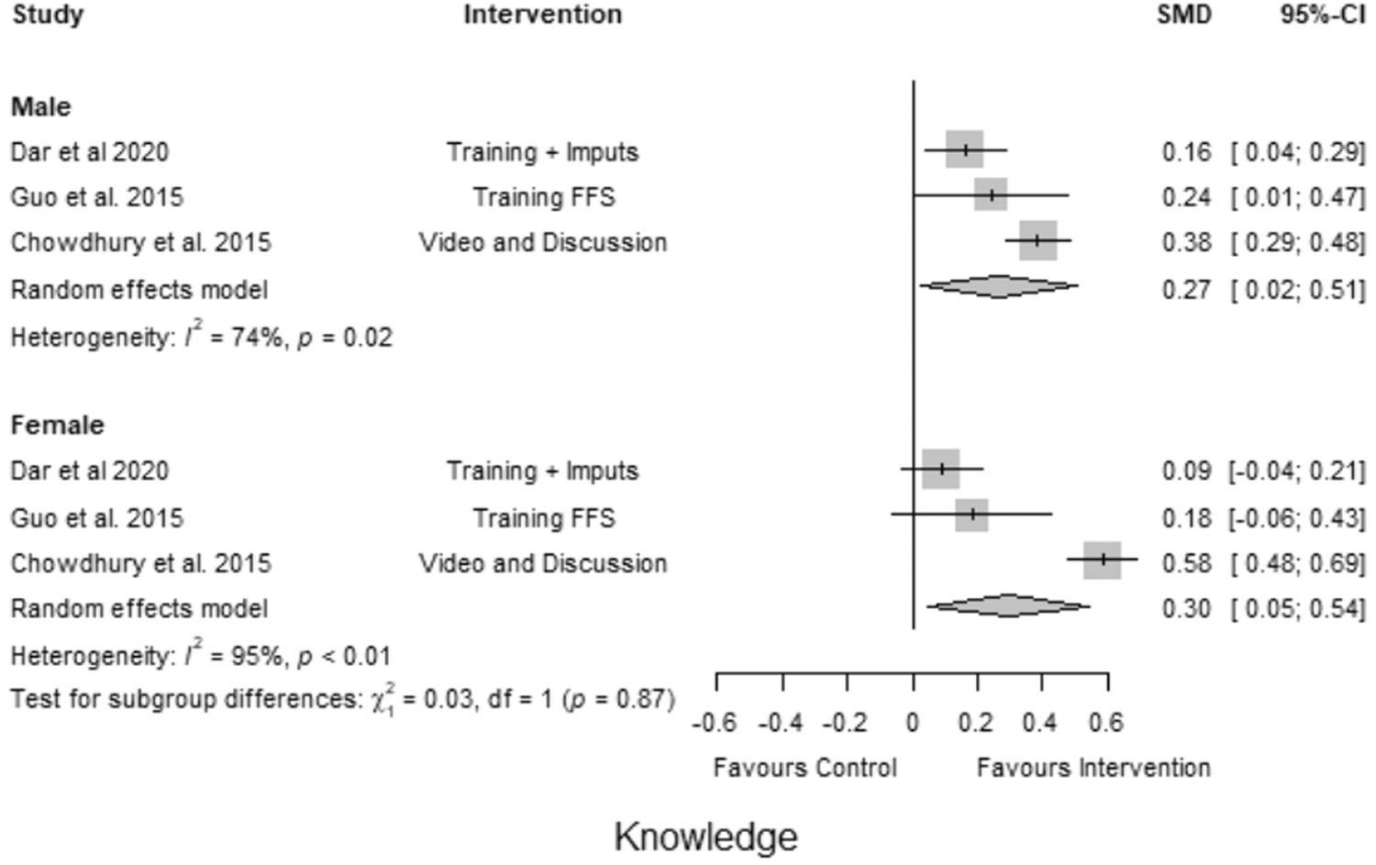
Subgroup analysis: Knowledge outcome.

In Figure 8, the effect on knowledge is plotted by group. The plot suggests that the interventions had varying effects on knowledge of CSA practices between males and females, with interventions having a consistently higher impact on females across all studies. The pooled effect is marginally higher for women farmers (SMD = 0.30, *I* ^2^ = 95%) as compared to men farmers (SMD = 0.27, *I* ^2^ = 74%), which implies that, on average, women farmers benefited more from the training interventions. However, the treatment effect estimates for each subgroup (males and females) were uncertain due to substantial heterogeneity (males: 74%; females: 95%).

**Figure 9:**
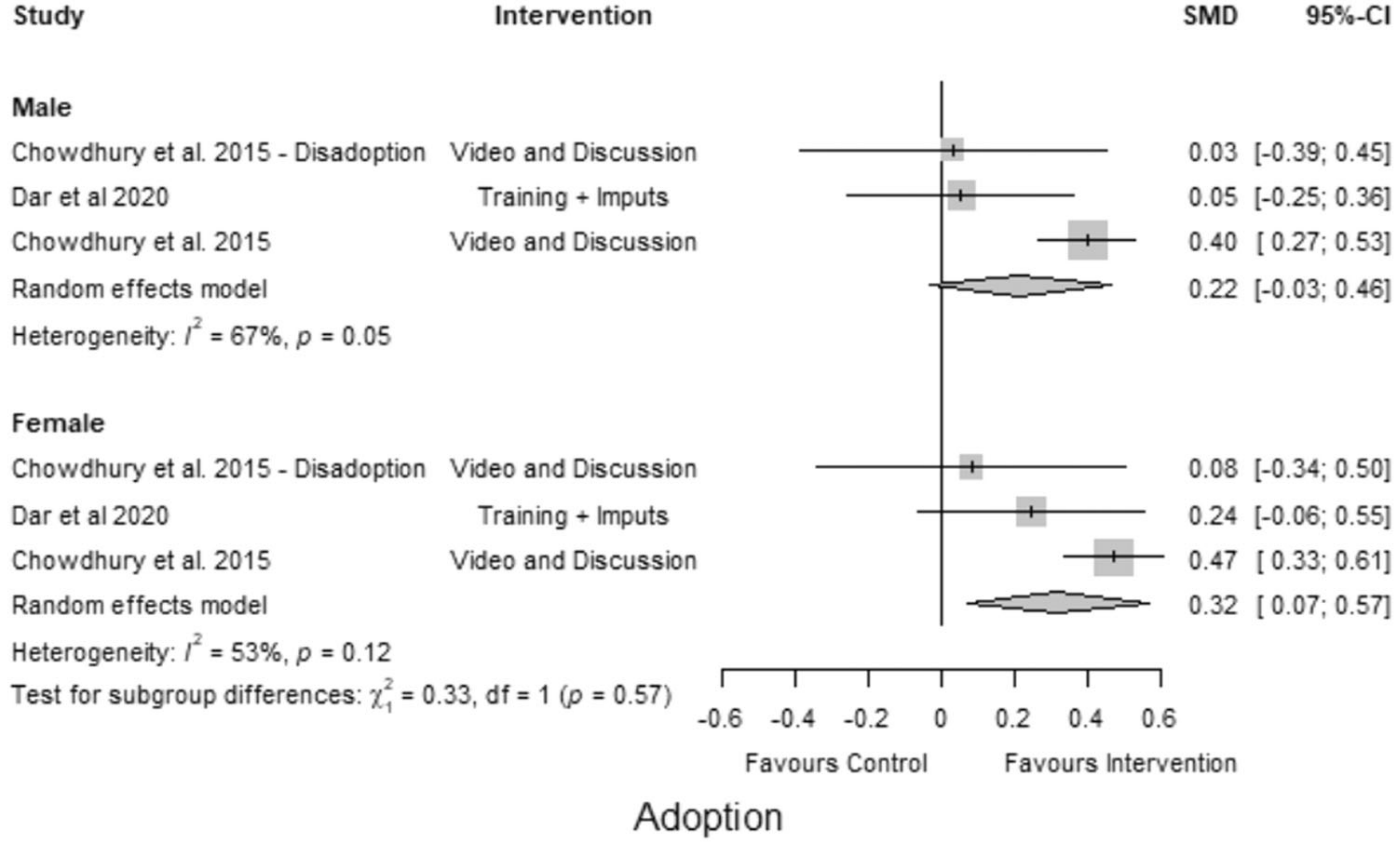
Subgroup analysis: Adoption of climate‐smart agriculture (CSA) practices.

Figure 9 illustrates a similar model for adoption practices. Training had a consistently greater effect on women farmers across all four studies. Interventions had varying effects on the adoption of CSA practices between women and men farmers, with training having a marginally higher impact on women farmers (SMD = 0.32) as compared to men farmers (SMD = 0.22). However, the high heterogeneity indicates that the results are inconsistent across trials, making the treatment effect estimates uncertain.

**Figure 10:**
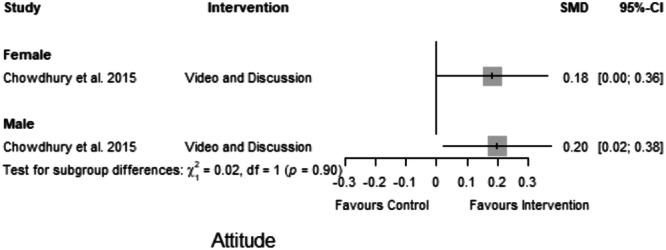
Subgroup analysis: Attitude.

Lastly, we obtained gender‐disaggregated data for attitude (Figure 10). The results for both men (SMD = 0.20, CI = 0.02 to 0.38) and women (SMD = 0.18, CI = 0.00 to 0.36) farmers showed little or no group differences and were insignificant in both studies.

While the existing evidence suggests that the interventions promoting CSA approaches appear to benefit women farmers more in terms of knowledge and adoption of CSA practices, the substantial heterogeneity emphasizes the need for cautious interpretation. Further research is warranted to understand the underlying factors contributing to these gender differences.

#### 6.4.6 Publication bias

**Figure 11:**
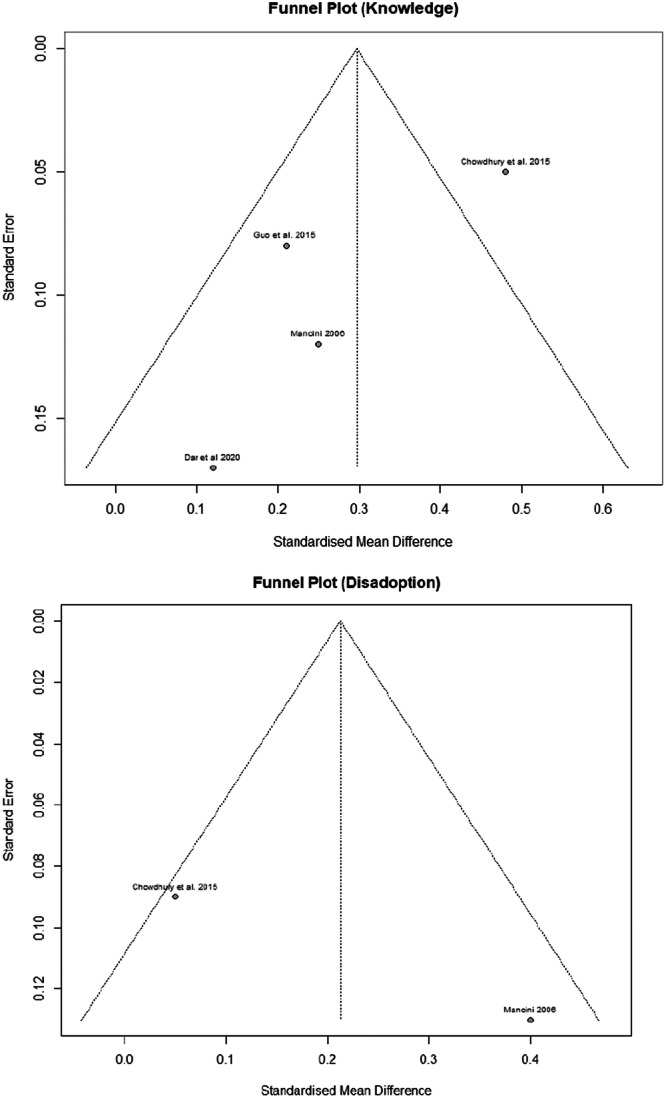
Funnel plot: Knowledge and disadoption.

**Figure 12:**
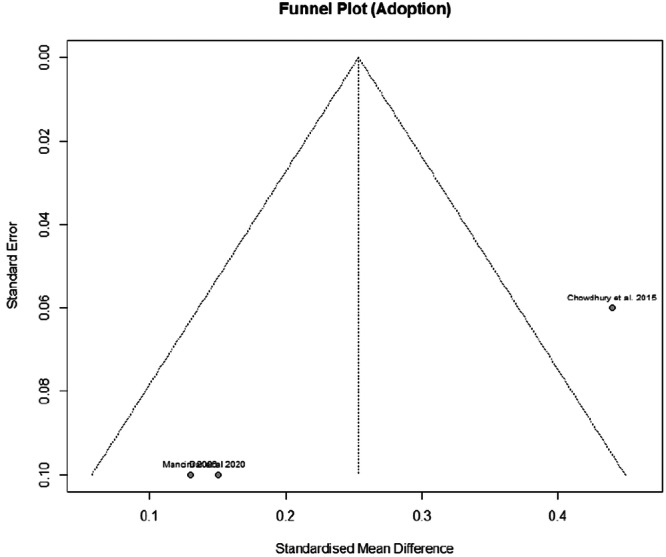
Funnel plot: Adoption.

In addition to the principal limitation of small sample sizes across the studies under review, further constraints are evident. There is a notable degree of heterogeneity within the study results, particularly pronounced in the context of adoption (*I*² = 81%) and disadoption (*I*² = 80%) outcomes. A further considerable limitation pertains to potential publication bias. Owing to the restricted sample sizes, it was not feasible to conclusively determine the presence of publication bias for the majority of the studies. Nevertheless, the knowledge outcomes do not appear to be subject to publication bias, as there is no apparent correlation between the effect size and increased standard error, an indicator that is typically suggestive of bias. This observation similarly applies to the adoption practices outcomes, where studies with less precise estimates do not appear to inflate the effect magnitude. However, the modesty of sample sizes precludes a definitive confirmation of publication bias. Moreover, the analysis of disadoption outcomes is based on a mere two studies, thus precluding a robust assessment of publication bias for this particular measure. (Figures 11 and 12).

## 7 DISCUSSION

### 7.1 Summary of main results

Drawing upon eight experimental and quasi‐experimental studies, this systematic review appraised interventions designed to bolster resilience via CSA practices among women farmers. A rigorous and expansive search strategy was employed, encompassing academic and online databases, citation tracking of pertinent studies, and consultations with subject matter experts. The analysis computed effect sizes for a range of outcomes, which included knowledge, attitudes, disadoption (of detrimental practices), adoption (of favorable practices), time utilization, and yield.

Geographically, the studies were distributed: four focused on South Asia, three on sub‐Saharan Africa—Ghana, Kenya, Tanzania, and Uganda are represented—and one hailing from China, indicative of the East Asia and Pacific region. In South Asia, the research was conducted within Bangladesh and India.

The scope of interventions scrutinized within these studies was confined to methods of knowledge transmission and capacity enhancement, encompassing social networking, peer learning, utilization of information and communication technologies, both group and individual training with demonstrations, agricultural extension services, and variants of farmer field schools. For instance, Mancini (2006) detailed the formation of IPM FFS in India's cotton belt, aiming to impart knowledge and hands‐on training concerning IPM techniques. Osumba et al. (2021) centered on the initiation of FFS that embraced innovative, climate‐resilient agribusiness practices. The “Farms of the Future” approach was employed by Nyasimi et al. (2017) to foster the uptake of climate‐smart agricultural technologies. Sharma et al. (2019) implemented a social marketing initiative to advocate for sustainable water use in Punjab, whereas Chowdhury et al. (2015) and Dar et al. (2020) leveraged video‐mediated training to propagate knowledge pertaining to botanical pesticide innovations in Bangladesh. Notably, there was an absence of studies evaluating other CSA promotion interventions, such as financial instruments (including credit and subsidies), institutional frameworks (e.g., collectivization through cooperatives, contract farming), land titling, communal infrastructure (e.g., irrigation dams), and community‐led natural resource management. This highlights a significant lacuna and underscores the necessity for further research and evidence generation in these domains.

Our review underscores the positive impact of knowledge dissemination interventions such as FFS and agriculture extension services on effectively improving farmers' knowledge and adoption of pest management, climate‐resilient rice seed varieties (STRVs), and the use of botanical pesticides. Specifically, three included studies (Chowdhury et al., 2015; Guo et al., 2015; Mancini, 2006) found a positive effect of knowledge dissemination and capacity‐building approaches, including farmer field schools and video‐mediated learning about knowledge and awareness of IPM. One study (Dar et al., 2020) reported a positive effect on farmers' knowledge and adoption of climate‐resilient rice varieties or STRVs. These studies demonstrate that the knowledge dissemination and capacity‐building approaches effectively increase farmers' knowledge and adoption of IPM. Furthermore, the study that reported a positive effect suggests that these approaches effectively increase farmers' knowledge and adoption of climate‐resilient rice varieties or STRVs. The adoption of IPM and botanical pesticides was studied in three studies (Chowdhury et al., 2015; Guo et al., 2015; Mancini, 2006). The paper by Guo et al. (2015) focuses on immediate changes in farmers' knowledge acquisition after accessing FFS training. It does not examine the impacts on behavior in the short term but rather the formation of knowledge for different components of the integrated technology package. The impact of adopting CSA practices on the use of time has been reported by one study (Mancini, 2006). According to the author, such adoption did not increase total labor time but did result in a more substantial contribution from women to the labor force.

Our findings align with previous studies. For instance, Smith et al. (2018) reported similar positive effects of FFS on knowledge transfer. However, their study also highlighted challenges related to scalability and long‐term sustainability. Waddington et al. (2014) and van den Berg et al. (2015) independently corroborated our results. Their studies highlighted the effectiveness of FFS in enhancing farmers' knowledge and promoting the adoption of beneficial practices. Notably, FFS played a pivotal role in reducing the overuse of pesticides—a critical concern for sustainable farming practices worldwide. These findings underscore the global relevance of FFS as a powerful mechanism for transferring specialist knowledge and empowering farmers. Our study echoes the work of Kpadonou et al. (2017) emphasizing the positive correlation between agricultural training and technology adoption. As farmers acquire knowledge and skills through training programs, they are better equipped to embrace innovative practices. The adoption of climate‐resilient technologies, such as improved seed varieties and pest management techniques, hinges on effective training strategies.

Interestingly, gender emerged as a nuanced factor in the adoption of CSA practices. While both men and women benefited from knowledge dissemination, marginal differences were observed. Women farmers exhibited slightly higher adoption rates, emphasizing the need for gender‐specific tailoring of interventions. Gender‐responsive approaches should consider social norms, access to resources, and decision‐making dynamics within farming households. Our findings align with those of Johnson and Lee (2019), who emphasized the role of extension services in promoting CSA practices among smallholder farmers. Their review echoed our observation that gender considerations play a crucial role in adoption outcomes. However, we find no effects on disadoption of harmful practices such as the overuse of pesticides. Only single studies identified outcomes of attitude and time use.

The results are heterogeneous and have moderate to high bias risk. The heterogeneity observed in our review warrants caution. Variability across studies may stem from contextual differences, study designs, or implementation fidelity. Additionally, the moderate to high risk of bias underscores the need for rigorous study designs and transparent reporting.

In conclusion, while knowledge dissemination through FFS and extension services holds promise, addressing disadoption challenges and tailoring interventions to gender‐specific needs remain critical for advancing sustainable agricultural methodologies.

#### 7.1.1 Quality of evidence

The experimental and quasi‐experimental evidence varied in quality, and the proportions of studies with high and moderate risks of bias were high (six out of eight studies).

Methodological details were generally reported poorly, which made it challenging to assess inclusion, assess risks of bias, and calculate effect sizes. Only two studies were evaluated to be of high confidence; the six others had a high risk of bias and low confidence, which indicated methodological limitations. In many studies, no serious attempts were made to control for confounding through statistical matching or other statistical analysis methods. Furthermore, in many cases, statistical significance tests were not conducted. Calculating standardized effect sizes from some experimental and quasi‐experimental studies was challenging due to the limited nature of reporting. The clear reporting of outcome data, standard deviations, and sample sizes for treatment and control groups at the end line in experimental and quasi‐experimental studies will aid in their inclusion in systematic reviews.

### 7.2 Overall completeness and applicability of evidence

We found eight experimental and quasi‐experimental studies looking at primary and secondary outcomes. Overall, this represents limited evidence on interventions promoting climate‐smart interventions. We found as few as two or three studies to provide results for some outcomes (e.g., attitude, yield, and decision‐making). We found no more than five studies that examine a common outcome. This is especially important when one considers the mixed quality of the evidence base and the relatively high number of high risks of bias experimental and quasi‐experimental studies. Geographically, the studies included are highly concentrated in a few countries in sub‐Saharan Africa and South Asia. The studies also focus largely on knowledge dissemination and capacity‐building approaches; there was a lack of studies on behavior and social change communication or institutional arrangements. Finally, as outlined earlier in the discussion, the evidence base is minimal regarding geographical coverage, other types of intervention and equity aspects.

### 7.3 Potential biases in the review process

Potential bias may be introduced about the lack of gray literature included in the review and the absence of non‐English literature. The first limitation is that the absence of literature published in languages other than English may produce biased estimates of the scope of the literature, as Arabic, Chinese, Arabic, French, and Portuguese (such as those in South America and West Africa) may be missed if study results are not translated or published in English. Bias could also have been introduced when the research team had different ideas about relevant interventions and outcomes or understandings of CSA programming. To address this potential source of bias, all full‐text reviews and coding decisions were made by at least two researchers on the team to come to a consensus on whether an article should be included.

## 8 AUTHORS' CONCLUSIONS

### 8.1 Implications for practice and policy

Despite the methodological and programmatic limitations of this review, all included studies reported a positive impacts of interventions promoting CSA in improving farmer knowledge and attitude and helping smallholder farmers adopt better practices.

Decision‐makers and practitioners should use the findings to inform their decision‐making processes, focusing on providing training, technical assistance, and access to information on CSA approaches to smallhoder men and women farmers.

Knowledge‐sharing platforms and FFS can be crucial in disseminating best practices and facilitating peer‐to‐peer learning.

### 8.2 Implications for research

This review finds a relatively small evidence base of both experimental and quasi‐experimental studies. Many experimental and quasi‐experimental studies had a high risk of bias. Overall, there is a need for more and better confidence studies to inform policy and programming.

Studies included in this review are highly concentrated in a few countries. Research from a wider geographical spread of countries is needed to ascertain the effectiveness of interventions in different contexts.

*Robust evidence is needed* to fill critical research gaps, such as studies on:

- Evidence is needed to report a broader range of interventions, such as financial incentives and institutional arrangements, which are critical in enhancing the adoption of CSA approaches.
- Long‐term impacts of adopting CSA approaches to determine how these interventions can contribute to long‐term resilience and food security are needed.
- Sex‐disaggregated data and research evaluating programs explicitly targeting women farmers with a focus on how power relations, food security and inequity differentially impact women farmers are needed.

## CONTRIBUTIONS OF AUTHORS

- Content: Ranjitha Puskur, Avni Mishra, Hugh Sharma Waddington, Ashrita Saran, and Sabina Singh are responsible for the content. Hugh Sharma Waddington is also the technical lead for the review.
- Systematic review methods: Ashrita Saran, Neha Gupta, Sujata Shirodkar and Hugh Sharma Waddington are responsible for systematic review methods.
- Statistical analysis: Hugh Sharma Waddington, Ashrita Saran, and Neha Gupta will conduct effect size extraction and statistical analysis.
- Information retrieval: Ashrita Saran is responsible for information retrieval based on searches designed by Sarah Young, Information Retrieval Specialist, Carnegie Mellon University, USA.

## DECLARATIONS OF INTEREST

The authors declare no conflicts of interest.

### 1 Plans for updating this review

We have no plans to update the review now, and it will depend on the available funding.

## SOURCES OF SUPPORT

### Internal sources

- New Source of support. Other

None.

### External sources

- This is funded by The CGIAR Generating Evidence and New Directions for Equitable Results(GENDER) Platform form. Other

This is funded by The CGIAR Generating Evidence and New Directions for Equitabl e Results (GENDER) Platform form.

## DIFFERENCES BETWEEN PROTOCOL AND REVIEW

We note no change between published protocol and the review.

## Supporting information

Supporting information.

## References

[cl21426-bib-0002] * Chowdhury, A. , Odame, H. H. , Thompson, S. , & Hauser, M. (2015). Enhancing farmers' capacity for botanical pesticide innovation through video‐mediated learning in Bangladesh. International Journal of Agricultural Sustainability, 13(4), 326–349. 10.1080/14735903.2014.997461

[cl21426-bib-0003] Dar, M. H. , Waza, S. A. , Nayak, S. , Chakravorty, R. , Zaidi, N. W. , & Hossain, M. (2020). Gender focused training and knowledge enhances the adoption of climate resilient seeds. Technology in Society, 63, 101388. 10.1016/j.techsoc.2020.101388 33250546 PMC7677893

[cl21426-bib-0004] Djido, A. , Zougmoré, R. B. , Houessionon, P. , Ouédraogo, M. , Ouédraogo, I. , & Diouf, N. S. (2021). To what extent do weather and climate information services drive the adoption of climate‐smart agriculture practices in Ghana? Climate Risk Management, 32, 100309. 10.1016/j.crm.2021.100309

[cl21426-bib-0005] Guo, M. , Jia, X. , Huang, J. , Kumar, K. B. , & Burger, N. E. (2015). Farmer field school and farmer knowledge acquisition in rice production: Experimental evaluation in China. Agriculture, Ecosystems & Environment, 209, 100–107. 10.1016/j.agee.2015.02.011

[cl21426-bib-0006] Mancini, F. (2006). *Impact of integrated pest management farmer field schools on health, farming systems, the environment, and livelihoods of cotton growers in Southern India* [Doctoral Thesis, Biological Farming Systems Group, Wageningen University and Research].

[cl21426-bib-0007] Nyasimi, M. , Kimeli, P. , Sayula, G. , Radeny, M. , Kinyangi, J. , & Mungai, C. (2017). Adoption and dissemination pathways for climate‐smart agriculture technologies and practices for climate‐resilient livelihoods in Lushoto, Northeast Tanzania. Climate, 5(3), 63. 10.3390/cli5030063

[cl21426-bib-0008] Osumba, J. J. , Recha, J. W. , & Oroma, G. W. (2021). Transforming agricultural extension service delivery through innovative bottom–up climate‐resilient agribusiness farmer field schools. Sustainability, 13(7), 3938. 10.3390/su13073938

[cl21426-bib-0009] Sharma, P. , Kaur, L. , Mittal, R. , Kaur, S. , & Kaur, S. (2019). Social marketing approach to bring change in water use behaviour of rural people of Punjab, India. Journal of Water and Climate Change, 10(4), 968–976. 10.2166/wcc.2018.150

[cl21426-bib-0011] Ado, A. M. , Savadogo, P. , & Abdoul‐Azize, H. T. (2019). Livelihood strategies and household resilience to food insecurity: Insight from a farming community in Aguie district of Niger. Agriculture and Human Values, 36(4), 747–761.

[cl21426-bib-0012] Aggarwal, P. K. , Jarvis, A. , Campbell, B. M. , Zougmoré, R. B. , Khatri‐Chhetri, A. , Vermeulen, S. J. , Loboguerrero, A. , Sebastian, L. S. , Kinyangi, J. , Bonilla‐Findji, O. , Radeny, M. , Recha, J. , Martinez‐Baron, D. , Ramirez‐Villegas, J. , Huyer, S. , Thornton, P. , Wollenberg, E. , Hansen, J. , Alvarez‐Toro, P. … Tan Yen, B. (2018). The climate‐smart village approach: Framework of an integrative strategy for scaling up adaptation options in agriculture. Ecology and Society, 23(1), 14.

[cl21426-bib-0013] Berrang‐Ford, L. , Siders, A. R. , Lesnikowski, A. , Fischer, A. P. , Callaghan, M. W. , Haddaway, N. R. , & Abu, T. Z. (2021). A systematic global stocktake of evidence on human adaptation to climate change. Nature Climate Change, 11(11), 989–1000.

[cl21426-bib-0014] Bui, L. V. , & Vu, T. B. (2020). A systematic review of climate‐smart agriculture (CSA) practices and its potential for adoption in the implementation of Nong thon moi in the 2021–2030 Strategy. CGSpace Repository of Agricultural Research Outputs.

[cl21426-bib-0015] Campbell, B. M. , Vermeulen, S. J. , Aggarwal, P. K. , Corner‐Dolloff, C. , Girvetz, E. , Loboguerrero, A. M. , & Wollenberg, E. (2016). Reducing risks to food security from climate change. Global Food Security, 11, 34–43.

[cl21426-bib-0016] Chanana‐Nag, N. , & Aggarwal, P. K. (2020). Woman in agriculture, and climate risks: Hotspots for development. Climatic Change, 158(1), 13–27. 10.1007/s10584-018-2233-z

[cl21426-bib-0017] Cumpston, M. , Li, T. , Page, M. J. , Chandler, J. , Welch, V. A. , Higgins, J. P. , & Thomas, J. (2019). Updated guidance for trusted systematic reviews: A new edition of the Cochrane Handbook for Systematic Reviews of Interventions. Cochrane Database Syst Rev, 10(ED000142), 14651858. 10.1002/14651858.ED000142 PMC10284251 31643080

[cl21426-bib-0086] Cutter, S. L. , Barnes, L. , Berry, M. , Burton, C. , Evans, E. , Tate, E. , & Webb, J. (2008). A place‐based model for understanding community resilience to natural disasters. Global Environmental Change, 18(4), 598–606.

[cl21426-bib-0018] Desai, B. H. , & Mandal, M. (2021). Role of climate change in exacerbating sexual and gender‐based violence against women: A new challenge for international law. Environmental Policy and Law, 51(3), 137–157.

[cl21426-bib-0019] Egger, M. , Davey, S. G. , Schneider, M. , & Minder, C. (1997). Bias in meta‐analysis detected by a simple, graphical test. BMJ, 315(7109), 629–634. 9310563 10.1136/bmj.315.7109.629 PMC2127453

[cl21426-bib-0020] Elbehri, A. , Challinor, A. , Verchot, L. , Angelsen, A. , Hess, T. , Ouled Belgacem, A. , Clark, H. , Badraoui, M. , Cowie, A. , De Silva, S. , Erickson, J. , Joar Hegland, S. , Iglesias, A. , Inouye, D. , Jarvis, A. , Mansur, E. , Mirzabaev, A. , Montanarella, L. , Murdiyarso, D. , … Walker, R. (2017). FAO‐IPCC expert meeting on climate change, land use and food security. FAO and IPCC. https://mel.cgiar.org/reporting/download/hash/TiDHMPZ2

[cl21426-bib-0021] FAO and Care . (2019). Good practices for integrating gender equality and women's empowerment in climate‐smart agriculture programmes. Food and Agriculture Organisation of United Nations. http://www.fao.org/3/ca3883en/ca3883en.pdf

[cl21426-bib-0023] Food and Agriculture Organization of the United Nations (FAO) . (2010). “Climate‐Smart” Agriculture: Policies, Practices and Financing for Food Security, Adaptation and Mitigation. Food and Agriculture Organization of the United Nations (FAO).

[cl21426-bib-0024] FAO . (2012). Sourcebook on climate‐smart agriculture, forestry and fisheries. Food and Agriculture Organisation of United Nations.

[cl21426-bib-0025] FAO . (2013). Climate smart agriculture: Sourcebook. Food and Agriculture Organisation of United Nations.

[cl21426-bib-0026] FAO . (2014). The state of food and agriculture report. In Innovation in family farming. FAO.

[cl21426-bib-0028] Gonzalez, P. C. , Shisler, S. , Moratti, M. , Yavuz, C. , Acharya, A. , Eyers, J. , & Snilstveit, B. (2021). Aquaculture for improving productivity, income, nutrition and women's empowerment in low‐and middle‐income countries: A systematic review and meta‐analysis. Campbell Systematic Reviews, 17(4), e1195. 37018454 10.1002/cl2.1195 PMC8988765

[cl21426-bib-0029] Gumucio, T. , Hansen, J. , Huyer, S. , & Van Huysen, T. (2020). Gender‐responsive rural climate services: A review of the literature. Climate and Development, 12(3), 241–254. 10.1080/17565529.2019.1613216

[cl21426-bib-0031] Hasegawa, T. , Sakurai, G. , Fujimori, S. , Takahashi, K. , Hijioka, Y. , & Masui, T. (2021). Extreme climate events increase risk of global food insecurity and adaptation needs. Nature Food, 2(8), 587–595. https://www.nature.com/articles/s43016-021-00335-4 37118168 10.1038/s43016-021-00335-4

[cl21426-bib-0032] Howland, F. , Le Coq, J. F. , & Acosta, M. (2019). Gender integration in agriculture, food security and climate change policy: A framework proposal. CGIAR (CCAFS Reports), 35. https://agritrop.cirad.fr/593757/

[cl21426-bib-0033] Huyer, S. , & Partey, S. (2020). Weathering the storm or storming the norms? Moving gender equality forward in climate‐resilient agriculture. CGIAR, 158(1), 1–12.

[cl21426-bib-0035] Shukla, P. R. , Skea, J. , Calvo Buendia, E. , Masson‐Delmotte, V. , Pörtner, H. O. , Roberts, D. C. , & Malley, J. (2019). Climate Change and Land: An IPCC special report on climate change, desertification, land degradation, sustainable land management, food security, and greenhouse gas fluxes in terrestrial ecosystems. IPCC.

[cl21426-bib-0036] IPCC . (2021). Mitigation of climate change. *Contribution of working group III to the fifth assessment report of the intergovernmental panel on climate change* . WG III contribution to the Sixth Assessment Report, 1453, 147. Intergovernmental Panel on Climate Change.

[cl21426-bib-0037] IRRI and CRISP . (2020). Training Module on Enabling Extension and Advisory Services (EAS) for Climate Smart Agriculture (CSA). International Rice Research Institute and Hyderabad, India, Centre for Research on Innovation and Science Policy.

[cl21426-bib-0039] Johnson, M. W. , & Lee, S. (2019). Extension services and climate‐smart agriculture: A systematic review. Agricultural Systems, 176, 102656. 10.1016/j.agsy.2019.102656

[cl21426-bib-0040] Jost, C. , Kyazze, F. , Naab, J. , Neelormi, S. , Kinyangi, J. , Zougmore, R. , Aggarwal, P. , Bhatta, G. , Chaudhury, M. , Tapio‐Bistrom, M. L. , Nelson, S. , & Kristjanson, P. (2016). Understanding gender dimensions of agriculture and climate change in smallholder farming communities. Climate and Development, 8(2), 133–144.

[cl21426-bib-0041] Khatri‐Chhetri, A. , Regmi, P. P. , Chanana, N. , & Aggarwal, P. K. (2020). Potential of climate‐smart agriculture in reducing women farmers' drudgery in high climatic risk areas. Climatic Change, 158(1), 29–42.

[cl21426-bib-0042] Korth, M. , Stewart, R. , Langer, L. , Madinga, N. , Rebelo Da Silva, N. , Zaranyika, H. , & de Wet, T. (2014). What are the impacts of urban agriculture programs on food security in low and middle‐income countries: A systematic review. Environmental Evidence, 3(1), 1–10.

[cl21426-bib-0043] Kpadonou, R. A. B. , Owiyo, T. , Barbier, B. , Denton, F. , Rutabingwa, F. , & Kiema, A. (2017). Advancing climate‐smart‐agriculture in developing drylands: Joint analysis of the adoption of multiple on‐farm soil and water conservation technologies in West African Sahel. Land Use Policy, 61, 196–207. https://www.sciencedirect.com/science/article/abs/pii/S0264837716300187

[cl21426-bib-0044] Lacombe, C. , Couix, N. , & Hazard, L. (2018). Designing agroecological farming systems with farmers: A review. Agricultural Systems, 165, 208–220. https://www.sciencedirect.com/science/article/abs/pii/S0308521X1830060X

[cl21426-bib-0045] Langan, D. , Higgins, J. P. , Jackson, D. , Bowden, J. , Veroniki, A. A. , Kontopantelis, E. , & Simmonds, M. (2019). A comparison of heterogeneity variance estimators in simulated random‐effects meta‐analyses. Research Synthesis Methods, 10(1), 83–98. 30067315 10.1002/jrsm.1316

[cl21426-bib-0046] Lawry, S. , Samii, C. , Hall, R. , Leopold, A. , Hornby, D. , & Mtero, F. (2017). The impact of land property rights interventions on investment and agricultural productivity in developing countries: A systematic review. Journal of Development Effectiveness, 9(1), 61–81.

[cl21426-bib-0047] Leisher, C. , Temsah, G. , Booker, F. , Day, M. , Samberg, L. , Prosnitz, D. , & Wilkie, D. (2016). Does the gender composition of forest and fishery management groups affect resource governance and conservation outcomes? A systematic map. Environmental Evidence, 5(6), 1–10.

[cl21426-bib-0048] Littell, J. H. (2018). Conceptual and practical classification of research reviews and other evidence synthesis products. Campbell Systematic Reviews, 14(1), 1–21. 10.4073/cmdp.2018.1 PMC8428026 37131386

[cl21426-bib-0049] Lopez‐Avila, D. , Husain, S. , Bhatia, R. , Nath, M. , & Vinaygyam, R. (2017). Agricultural innovation: An evidence gap map (3ie Evidence Gap Map Report 12) (p. 12). International Initiative for Impact Evaluation (3ie).

[cl21426-bib-0050] Mafongoya, P. L. , Bationo, A. , Kihara, J. , & Waswa, B. S. (2006). Appropriate technologies to replenish soil fertility in southern Africa. Nutrient Cycling in Agroecosystems, 76(2), 137–151.

[cl21426-bib-0051] Makate, C. (2019). Effective scaling of climate smart agriculture innovations in African smallholder agriculture: A review of approaches, policy and institutional strategy needs. Environmental Science & Policy, 96, 37–51. https://www.sciencedirect.com/science/article/abs/pii/S1462901118309377

[cl21426-bib-0053] Mbow, C. , Rosenzweig, C. , Barioni, L. G. , Benton, T. G. , Herrero, M. , Krishnapillai, M. , Liwenga, E. , Pradhan, P. , Rivera‐Ferre, M. G. , Sapkota, T. Tubiello, F. N. , & Xu, Y. (2019). Food security. In P. R. Shukla , J. Skea , E. Calvo Buendia , V. Masson‐Delmotte , H.‐O. Pörtner , D. C. Roberts , P. Zhai , R. Slade , S. Connors , R. van Diemen , M. Ferrat , E. Haughey , S. Luz , S. Neogi , M. Pathak , J. Petzold , J. Portugal Pereira , P. Vyas , E. Huntley , K. Kissick , M. Belkacemi , & J. Malley (Eds.), Climate change and land: An IPCC special report on climate change, desertification, land degradation, sustainable land management, food security, and greenhouse gas fluxes in terrestrial ecosystems. IPCC special report. (In press).

[cl21426-bib-0055] Munoz Boudet, A. M. , Buitrago, P. , Leroy De La Briere, B. , Newhouse, D. L. , Rubiano Matulevich, E. C. , Scott, K. , & Suarez‐Becerra, P. (2018). Gender differences in poverty and household composition through the life‐cycle: A global perspective. World Bank Policy Research Working Paper No. 8360. https://papers.ssrn.com/sol3/papers.cfm?abstract_id=3135590

[cl21426-bib-0058] Nhat Lam Duyen, T. , Rañola, R. F. , Sander, B. O. , Wassmann, R. , Tien, N. D. , & Ngoc, N. N. K. (2021). A comparative analysis of gender and youth issues in rice production in North, Central, and South Vietnam. Climate and Development, 13(2), 115–127.

[cl21426-bib-0087] Nyasimi, M. K. (2007). Transforming lands and livelihoods in the Awach River Basin of Lake Victoria, western Kenya. Iowa State University.

[cl21426-bib-0059] O'Neill, J. , Tabish, H. , Welch, V. , Petticrew, M. , Pottie, K. , Clarke, M. , & Tugwell, P. (2014). Applying an equity lens to interventions: Using PROGRESS ensures consideration of socially stratifying factors to illuminate inequities in health. Journal of Clinical Epidemiology, 67(1), 56–64. 24189091 10.1016/j.jclinepi.2013.08.005

[cl21426-bib-0060] Paavola, J. , & Adger, W. N. (2006). Fair adaptation to climate change. Ecological Economics, 56(4), 594–609.

[cl21426-bib-0061] Paudyal, B. R. , Chanana, N. , Khatri‐Chhetri, A. , Sherpa, L. , Kadariya, I. , & Aggarwal, P. (2019). Gender integration in climate change and agricultural policies: The case of Nepal. Frontiers in Sustainable Food Systems, 3, 66. 10.3389/fsufs.2019.00066/full

[cl21426-bib-0062] Petheram, L. , Zander, K. K. , Campbell, B. M. , Chris, H. , & Natasha, S. (2010). Strange changes': Indigenous perspectives of climate change and adaptation in NE Arnhem Land (Australia). Global Environmental Change, 20(4), 681–692.

[cl21426-bib-0063] Portar, J. R. , Xie, L. , Challinor, A. J. , Cochrane, K. , Howden, S. M. , Iqbal, M. M. , & Travasso, M. I. (2014). Contribution of Working Group II to the fifth assessment report of the intergovernmental panel on climate chan. Cambridge University Press.

[cl21426-bib-0064] Rosenstock, T. S. , Lamanna, C. , Chesterman, S. , Bell, P. , Arslan, A. , Richards, M. , Rioux, J. , Akinleye, A. , Champalle, C. , & Cheng, Z. (2016). The scientific basis of climate‐smart agriculture: A systematic review protocol. CCAFS Working Paper no. 138. CGIAR Research Program on Climate Change, Agriculture and Food Security (CCAFS).

[cl21426-bib-0065] Rosenstock, T. S. , Lamanna, C. , Chesterman, S. , Bell, P. , Arslan, A. , Richards, M. B. , Rioux, J. , Akinleye, A. O. , Champalle, C. , & Cheng, Z. (2016). The scientific basis of climate‐smart agriculture: A systematic review protocol. CCAFS Working Paper.

[cl21426-bib-0066] Roudier, P. , Muller, B. , d'Aquino, P. , Roncoli, C. , Soumaré, M. A. , Batté, L. , & Sultan, B. (2014). The role of climate forecasts in smallholder agriculture: Lessons from participatory research in two communities in Senegal. Climate Risk Management, 2, 42–55.

[cl21426-bib-0067] Schut, M. , van Asten, P. , Okafor, C. , Hicintuka, C. , Mapatano, S. , Nabahungu, N. L. , Kagabo, D. , Muchunguzi, P. , Njukwe, E. , & Dontsop‐Nguezet, P. M. (2016). Sustainable intensification of agricultural systems in the central African highlands: The need for institutional innovation. Agricultural Systems, 145, 165–176.

[cl21426-bib-0068] Sivabalan, K. C. , Ravichamy, P. , Ranganathan, T. T. , & Krishnan, J. (2021). Effectiveness of farmer field school and conventional extension trainings on knowledge gain among farm women. Asian Journal of Agricultural Extension, Economics & Sociology, 39(7), 96–103. 10.9734/ajaees/2021/v39i730613

[cl21426-bib-0069] Smith, J. K. , Brown, L. M. , & Garcia, R. (2018). Evaluating the impact of Farmer Field Schools on agricultural knowledge transfer. Journal of Sustainable Agriculture, 42(7), 654–670.

[cl21426-bib-0088] Stewart, A. M. , Grossman, L. , Nguyen, M. , Maximino, C. , Rosemberg, D. B. , Echevarria, D. J. , & Kalueff, A. V. (2014). Aquatic toxicology of fluoxetine: Understanding the knowns and the unknowns. Aquatic Toxicology, 156, 269–273. 25245382 10.1016/j.aquatox.2014.08.014

[cl21426-bib-0070] Teklewold, H. , Gebrehiwot, T. , & Bezabih, M. (2019). Climate smart agricultural practices and gender differentiated nutrition outcome: An empirical evidence from Ethiopia. World Development, 122, 38–53.

[cl21426-bib-0071] Tomayko, E. J. , Mosso, K. L. , Cronin, K. A. , Carmichael, L. , Kim, K. , Parker, T. , & Adams, A. K. (2017). Household food insecurity and dietary patterns in rural and urban American Indian families with young children. BMC Public Health, 17(1), 1–10. 28666476 10.1186/s12889-017-4498-y PMC5493116

[cl21426-bib-0072] Ton, G. , Vellema, W. , Desiere, S. , Weituschat, S. , & D'Haese, M. (2018). Contract farming for improving smallholder incomes: What can we learn from effectiveness studies? World Development, 104, 46–64.

[cl21426-bib-0073] Totin, E. , Segnon, A. C. , Schut, M. , Affognon, H. , Zougmoré, R. B. , Rosenstock, T. , & Thornton, P. K. (2018). Institutional perspectives of climate‐smart agriculture: A systematic literature review. Sustainability, 10(6), 1990.

[cl21426-bib-0074] UNDP . (2016). Gender equality in national climate action: Planning for genderresponsive nationally determined contribution. United Nations Development Programme.

[cl21426-bib-0075] Hatfield, J. , Takle, G. , Grotjahn, R. , Holden, P. , l zaurralde, R. C. , Mader, T. , Marshall, E. , & Liverman, D. (2014). Ch. 6: Agriculture. In J. M. Melillo , T. C. Richmond , & G. W. Yohe (Eds.), Climate change impacts in the United States: The third national climate assessment (pp. 150–174). Global Change Research Program. USGCRP.

[cl21426-bib-0089] USGCRP . (2018). Impacts, risks, and adaptation in the United States: Fourth national climate assessment (Vol. 2. p. 1515). In D. R. Reidmiller , C. W. Avery , D. R. Easterling , K. E. Kunkel , K. L. M. Lewis , T. K. Maycock , & B. C. Stewart (Eds.), U.S. Global Change Research Program. 10.7930/NCA4.2018

[cl21426-bib-0076] van den Berg, H. , Phillips, S. , Dicke, M. , & Fredrix, M. (2015). Impacts of farmer field schools in the human, social, natural and financial domain: A qualitative review. Food Security, 12(6), 1443–1459.

[cl21426-bib-0077] Waddington, H. , & Cairncross, S. (2021). Water, sanitation and hygiene for reducing childhood mortality in low‐and middle‐income countries. Campbell Systematic Reviews, 17(1), e1135. 37050969 10.1002/cl2.1135 PMC8356349

[cl21426-bib-0078] Waddington, H. , White, H. , Snilstveit, B. , Hombrados, J. G. , Vojtkova, M. , Davies, P. , & Tugwell, P. (2012). How to do a good systematic review of effects in international development: A tool kit. Journal of Development Effectiveness, 4(3), 359–387.

[cl21426-bib-0079] Waddington, H. , Snilstveit, B. , Hombrados, J. , Vojtkova, M. , Phillips, D. , Davies, P. , & White, H. (2014). Farmer field schools for improving farming practices and farmer outcomes: A systematic review. Campbell Systematic Reviews, 10(1), i335.

[cl21426-bib-0080] Waddington, H. , Ada, S. , Juliette, F. , Marie, G. , Denny, J. , & Jennifer, S. (2019). Citizen engagement in public services in low‐and middle‐income countries: A mixed‐methods systematic review of participation, inclusion, transparency and accountability (PITA) initiatives. Campbell Systematic Reviews, 15(1–2), e1025. 37131473 10.1002/cl2.1025 PMC8356537

[cl21426-bib-0081] Wester, M. , & Lama, P. D. (2019). Women as agents of change?: Reflections on women in climate adaptation and mitigation in the global north and the global South. In C. Kinnvall & H. Rydstrom (Eds.), Climate hazards, disasters, and gender ramifications (pp. 67–85). Routledge.

[cl21426-bib-0082] World Health Organization . (2016). Burning opportunity: Clean household energy for health, sustainable development, and wellbeing of women and children. WHO Library Cataloguing‐in‐Publication Data. https://www.who.int/about/licensing/copyright_form/en/index.html

[cl21426-bib-0090] World Bank, International Fund for Agricultural Development (IFAD) & Food and Agriculture Organization (FAO) . (2008). Gender in agriculture sourcebook. World Bank Publications.

[cl21426-bib-0083] World Bank, FAO & IFAD . (2015). Gender in climate‐smart agriculture: Module 18 for gender in agriculture source‐book. Agriculture global practice. World Bank Group.

