# Supplementary material for: Interventions promoting resilience through climate smart agricultural practices for women farmers: A systematic review
Source: Campbell Syst Rev. 2024 Aug 27;20(3):e1426. doi: 10.1002/cl2.1426 (PMC11347864; doi:10.1002/cl2.1426)
Supplement: Supplementary file 1 — Supporting information. [file CL2-20-e1426-s001.docx]

**Appendices**

**Appendix 1. Search Strategy**

Database name:Web of Science Core Collection(Social Sciences Citation Index (SSCI), Science Citation Index Expanded (SCI-EXPANDED), Conference Proceedings Citation Index – Science (CPCI-S), Conference Proceedings Citation Index – Social Science & Humanities (CPCI-SSH), Emerging Sources Citation Index (ESCI))

Platform: CAB Direct

Date searched: April 17, 2022

Number of results: 12,636

| 1 Agriculture/Farm | title:(farm* OR agricultur* OR smallhold* OR "small hold*" OR microfarm* OR micro-farm* OR pastoral* OR agroforest* OR agropastoral OR agro-pastoral OR ejido OR silvopastoral OR producer* OR grower* OR agronomy OR husbandry OR aquacultur* OR floricultur* OR horticultur* OR cultivat* OR dairy OR livestock OR crop*) OR ab:(farm* OR agricultur* OR smallhold* OR "small hold*" OR microfarm* OR micro-farm* OR pastoral* OR agroforest* OR agropastoral OR agro-pastoral OR ejido OR silvopastoral OR producer* OR grower* OR agronomy OR husbandry OR aquacultur* OR floricultur* OR horticultur* OR cultivat* OR dairy OR livestock OR crop*) OR de:(smallholders OR pastoral OR farm OR agriculture OR agronomy OR husbandry OR aquaculture OR floriculture OR horticulture OR dairy OR livestock OR crop OR farming) | 2,251,164 |
| --- | --- | --- |
| 2  Women | title:(gender* OR woman* OR women* OR mother* OR maternal OR female* OR wife* OR wives OR feminization OR feminisation) OR ab:(gender* OR woman* OR women* OR mother* OR maternal OR female* OR wife* OR wives OR feminization OR feminisation) OR de:("gender relations" OR women) | 737,395 |
| 3  Climate smart agricultural practices | title:("climate smart" OR ((Innovat* OR conservation) AND agricultur*) OR (climate AND (adapt* OR resilien*)) OR "greenhouse gas emission*" OR "GHGs emission*" OR "ghg emission*" OR "crop residue retention" OR (rotat* AND crop*) OR "site-specific nutrient management" OR "water management" OR "planting pit*" OR "rainwater harvesting" OR "water harvesting" OR "zero till*" OR "no till*" OR "minimum till*" OR mulch* OR "laser land levelling" OR terrace* OR bund* OR compost* OR "cover crop*" OR "high-yield*" OR "stress-toleran*" OR "drought-toleran*" OR "drought-resist*" OR "feed management" OR destocking OR ((adaptive OR improved) AND breed*) OR "pasture management" OR "integrated nutrient management" OR postharvest OR "post-harvest" OR "soil management" OR (rain* AND (fed OR feed*)) OR (trench* AND hill*) OR hilling OR (contour AND (bund* OR plough* OR plow*)) OR zai OR "water break*" OR intercrop* OR (companion AND (plant* OR variet* OR species)) OR "integrated soil fertility management" OR ISFM) OR ab:("climate smart" OR ((Innovat* OR conservation) AND agricultur*) OR (climate AND (adapt* OR resilien*)) OR "greenhouse gas emission*" OR "GHGs emission*" OR "ghg emission*" OR "crop residue retention" OR (rotat* AND crop*) OR "site-specific nutrient management" OR "water management" OR "planting pit*" OR "rainwater harvesting" OR "water harvesting" OR "zero till*" OR "no till*" OR "minimum till*" OR mulch* OR "laser land levelling" OR terrace* OR bund* OR compost* OR "cover crop*" OR "high-yield*" OR "stress-toleran*" OR "drought-toleran*" OR "drought-resist*" OR "feed management" OR destocking OR ((adaptive OR improved) AND breed*) OR "pasture management" OR "integrated nutrient management" OR postharvest OR "post-harvest" OR "soil management" OR (rain* AND (fed OR feed*)) OR (trench* AND hill*) OR hilling OR (contour AND (bund* OR plough* OR plow*)) OR zai OR "water break*" OR intercrop* OR (companion AND (plant* OR variet* OR species)) OR "integrated soil fertility management" OR ISFM) OR de:(no-tillage OR bunds OR "rainfed agriculture" OR "drought tolerance" OR "greenhouse gases" OR "soil management" OR "crop rotation" OR "water management" OR "water harvesting" OR "zero tillage" OR "contour cultivation" OR "companion crops") | 665,792 |
| 4  Financial approaches | title:(compensat* OR financ* OR insur* OR ((financ* OR monetary OR cash OR money) AND (assist* OR support* OR transfer* OR voucher* OR grant* OR payment*)) OR fund* OR subsid* OR *credit* OR "risk management" OR "risk mitigation" OR "village savings and loans association*" OR VSLA) OR ab:(compensat* OR financ* OR insur* OR ((financ* OR monetary OR cash OR money) AND (assist* OR support* OR transfer* OR voucher* OR grant* OR payment*)) OR fund* OR subsid* OR *credit* OR "risk management" OR "risk mitigation" OR "village savings and loans association*" OR VSLA) | 332,256 |
| 5  Institutional arrangements | title:("farmer* OR agricultur* near/3 (organization* OR organization*) OR contract farm*" OR "land titl*" OR collectiv* OR cooperative* OR "farmer* based organization*" OR "farmer* based organization*" OR "farmer* organisation*" OR "farmer* organization*" OR "farmer* group*" OR "community infrastructure" OR "irrigation dam*" OR "legislative reform*" OR "policy reform*") OR ab:("contract farm*" OR "land titl*" OR collectiv* OR cooperative* OR "farmer* based organization*" OR "farmer* based organization*" OR "farmer* organisation*" OR "farmer* organization*" OR "farmer* group*" OR "community infrastructure" OR "irrigation dam*" OR "legislative reform*" OR "policy reform*") OR de:("contract farming" OR "farmers' associations" OR "cooperative farm*") | 93,925 |
| 6  Behavioural and social change on gender norms | title:(women near/3 equal* OR promot* OR facilitat* OR motivat* OR encourag* OR advoca* OR persua* OR sustain* OR behaviour* OR behavior* OR habit* OR custom* OR tendenc* OR packag* OR program* OR campaign* OR educat* OR train* OR lectur* OR workshop* OR game* OR demonstrat* OR quiz*) AND title: gender AND (stereo* OR norm* OR equal* OR inequal* OR transform* OR equit* OR inequit*) | 166 |
| 7 | ab:(promot* OR facilitat* OR motivat* OR encourag* OR advoca* OR persua* OR sustain* OR behaviour* OR behavior* OR habit* OR custom* OR tendenc* OR packag* OR program* OR campaign* OR educat* OR train* OR lectur* OR workshop* OR game* OR demonstrat* OR quiz*) AND ab:("gender stereo*" OR "gender norm*" OR "gender role*" OR "gender equal*" OR "gender inequal*" OR "gender transform*" OR "gender equit*" OR "gender inequit*") | 3,618 |
| 8  Knowledge dissemination approaches | title:("information dissemination" OR "knowledge dissemination" OR "competency-based education" OR "culturally competent care" OR "consumer health information" OR "consumer advocacy" OR "knowledge management" OR "dissemination strategy" OR "knowledge uptake" OR "knowledge transfer" OR "direct particip* program*" OR "community mobilisation" OR "community mobilization" OR "community strengthen*" OR "organisation* develop*" OR "organization* develop*" OR "workforce develop*" OR "social market*" OR "social media" OR "communication technolog*" OR *phone* OR SMS OR "text messag*" OR television* OR radio* OR "social capital" OR "social network*" OR extension OR "demonstration plot*" OR "field day*" OR "field school*" OR "climate information service*" OR "weather information service*" OR "climate information system*" OR "weather information system*") OR ab:("information dissemination" OR "knowledge dissemination" OR "competency-based education" OR "culturally competent care" OR "consumer health information" OR "consumer advocacy" OR "knowledge management" OR "dissemination strategy" OR "knowledge uptake" OR "knowledge transfer" OR "direct particip* program*" OR "community mobilisation" OR "community mobilization" OR "community strengthen*" OR "organisation* develop*" OR "organization* develop*" OR "workforce develop*" OR "social market*" OR "social media" OR "communication technolog*" OR *phone* OR SMS OR "text messag*" OR television* OR radio* OR "social capital" OR "social network*" OR extension OR "demonstration plot*" OR "field day*" OR "field school*" OR "climate information service*" OR "weather information service*" OR "climate information system*" OR "weather information system*") OR de:(communication OR "cell phones" OR extension) | 271,485 |
| 9 | #3 OR #4 OR #5 OR #6 OR #7 OR #8 | 1,270,540 |
| 10  LMICs and Developing Countries | title:(afghanistan or albania or algeria or "american samoa" or angola or "antigua and barbuda" or antigua or barbuda or argentina or armenia or armenian or aruba or azerbaijan or bahrain or bangladesh or barbados or belarus or byelarus or belorussia or byelorussian or belize or "british honduras" or benin or dahomey or bhutan or bolivia or bosnia or herzegovina or botswana or bechuanaland or brazil or brasil or bulgaria or "burkina faso" or "burkina fasso" or "upper volta" or burundi or urundi or "cabo verde" or "cape verde" or cambodia or kampuchea or "khmer republic" or cameroon or cameron or cameroun or "central african republic" or "ubangi shari" or chad or chile or china or colombia or comoros or "comoro islands" or "iles comores" or mayotte or congo or zaire or "costa rica" or "cote d ivoire" or "cote divoire" or "cote d ivoire" or "ivory coast" or croatia or cuba or cyprus or "czech republic" or czechoslovakia or djibouti or "french somaliland" or dominica or "dominican republic" or ecuador or egypt or "united arab republic" or "el salvador" or eritrea or estonia or eswatini or swaziland or ethiopia or fiji or gabon or "gabonese republic" or gambia or "georgia (republic)" or georgian or ghana or "gold coast" or gibraltar or greece or grenada or guam or guatemala or guinea or guyana or "british guiana" or haiti or hispaniola or honduras or hungary or india or indonesia or timor or iran or iraq or "isle of man" or jamaica or jordan or kazakhstan or kazakh or kenya or korea or kosovo or kyrgyzstan or kirghizia or kirgizstan or "kyrgyz republic" or kirghiz or laos or "lao pdr" or "lao people's democratic republic" or latvia or lebanon or "lebanese republic" or lesotho or basutoland or liberia or libya or "libyan arab jamahiriya" or lithuania or macau or macao or macedonia or madagascar or "malagasy republic" or malawi or nyasaland or malaysia or "malay federation" or "malaya federation" or maldives or "indian ocean" or mali or malta or micronesia or kiribati or "marshall islands" or nauru or "northern mariana islands" or palau or tuvalu or mauritania or mauritius or mexico or moldova or moldovian or mongolia or montenegro or morocco or ifni or mozambique or "portuguese east africa" or myanmar or burma or namibia or nepal or "netherlands antilles" or nicaragua or niger or nigeria or oman or muscat or pakistan or panama or "papua new guinea" or "new guinea" or paraguay or peru or philippines or philipines or phillipines or phillippines or poland or "polish people's republic" or portugal or "portuguese republic" or "puerto rico" or romania or russia or "russian federation" or ussr or "soviet union" or "union of soviet socialist republics" or rwanda or ruanda or samoa or "pacific islands" or polynesia or "samoan islands" or "navigator island" or "navigator islands" or "sao tome and principe" or "saudi arabia" or senegal or serbia or seychelles or "sierra leone" or slovakia or "slovak republic" or slovenia or melanesia or "solomon island" or "solomon islands" or "norfolk island" or "norfolk islands" or somalia or "south africa" or "south sudan" or "sri lanka" or ceylon or "saint kitts and nevis" or "st. kitts and nevis" or "saint lucia" or "st. lucia" or "saint vincent and the grenadines" or "saint vincent" or "st. vincent" or grenadines or sudan or suriname or surinam or "dutch guiana" or "netherlands guiana" or syria or "syrian arab republic" or tajikistan or tadjikistan or tadzhikistan or tadzhik or tanzania or tanganyika or thailand or siam or "timor leste" or "east timor" or togo or "togolese republic" or tonga or "trinidad and tobago" or trinidad or tobago or tunisia or turkey or turkmenistan or turkmen or uganda or ukraine or uruguay or uzbekistan or uzbek or vanuatu or "new hebrides" or venezuela or vietnam or "viet nam" or "middle east" or "west bank" or gaza or palestine or yemen or yugoslavia or zambia or zimbabwe or "northern rhodesia" or "global south" or "africa south of the sahara" or "sub-saharan africa" or "subsaharan africa" or "africa, central" or "central africa" or "africa, northern" or "north africa" or "northern africa" or magreb or maghrib or sahara or "africa, southern" or "southern africa" or "africa, eastern" or "east africa" or "eastern africa" or "africa, western" or "west africa" or "western africa" or "west indies" or "indian ocean islands" or caribbean or "central america" or "latin america" or "south and central america" or "south america" or "asia, central" or "central asia" or "asia, northern" or "north asia" or "northern asia" or "asia, southeastern" or "southeastern asia" or "south eastern asia" or "southeast asia" or "south east asia" or "asia, western" or "western asia" or "europe, eastern" or "east europe" or "eastern europe") OR ab:(afghanistan or albania or algeria or "american samoa" or angola or "antigua and barbuda" or antigua or barbuda or argentina or armenia or armenian or aruba or azerbaijan or bahrain or bangladesh or barbados or belarus or byelarus or belorussia or byelorussian or belize or "british honduras" or benin or dahomey or bhutan or bolivia or bosnia or herzegovina or botswana or bechuanaland or brazil or brasil or bulgaria or "burkina faso" or "burkina fasso" or "upper volta" or burundi or urundi or "cabo verde" or "cape verde" or cambodia or kampuchea or "khmer republic" or cameroon or cameron or cameroun or "central african republic" or "ubangi shari" or chad or chile or china or colombia or comoros or "comoro islands" or "iles comores" or mayotte or congo or zaire or "costa rica" or "cote d ivoire" or "cote divoire" or "cote d ivoire" or "ivory coast" or croatia or cuba or cyprus or "czech republic" or czechoslovakia or djibouti or "french somaliland" or dominica or "dominican republic" or ecuador or egypt or "united arab republic" or "el salvador" or eritrea or estonia or eswatini or swaziland or ethiopia or fiji or gabon or "gabonese republic" or gambia or "georgia (republic)" or georgian or ghana or "gold coast" or gibraltar or greece or grenada or guam or guatemala or guinea or guyana or "british guiana" or haiti or hispaniola or honduras or hungary or india or indonesia or timor or iran or iraq or "isle of man" or jamaica or jordan or kazakhstan or kazakh or kenya or korea or kosovo or kyrgyzstan or kirghizia or kirgizstan or "kyrgyz republic" or kirghiz or laos or "lao pdr" or "lao people's democratic republic" or latvia or lebanon or "lebanese republic" or lesotho or basutoland or liberia or libya or "libyan arab jamahiriya" or lithuania or macau or macao or macedonia or madagascar or "malagasy republic" or malawi or nyasaland or malaysia or "malay federation" or "malaya federation" or maldives or "indian ocean" or mali or malta or micronesia or kiribati or "marshall islands" or nauru or "northern mariana islands" or palau or tuvalu or mauritania or mauritius or mexico or moldova or moldovian or mongolia or montenegro or morocco or ifni or mozambique or "portuguese east africa" or myanmar or burma or namibia or nepal or "netherlands antilles" or nicaragua or niger or nigeria or oman or muscat or pakistan or panama or "papua new guinea" or "new guinea" or paraguay or peru or philippines or philipines or phillipines or phillippines or poland or "polish people's republic" or portugal or "portuguese republic" or "puerto rico" or romania or russia or "russian federation" or ussr or "soviet union" or "union of soviet socialist republics" or rwanda or ruanda or samoa or "pacific islands" or polynesia or "samoan islands" or "navigator island" or "navigator islands" or "sao tome and principe" or "saudi arabia" or senegal or serbia or seychelles or "sierra leone" or slovakia or "slovak republic" or slovenia or melanesia or "solomon island" or "solomon islands" or "norfolk island" or "norfolk islands" or somalia or "south africa" or "south sudan" or "sri lanka" or ceylon or "saint kitts and nevis" or "st. kitts and nevis" or "saint lucia" or "st. lucia" or "saint vincent and the grenadines" or "saint vincent" or "st. vincent" or grenadines or sudan or suriname or surinam or "dutch guiana" or "netherlands guiana" or syria or "syrian arab republic" or tajikistan or tadjikistan or tadzhikistan or tadzhik or tanzania or tanganyika or thailand or siam or "timor leste" or "east timor" or togo or "togolese republic" or tonga or "trinidad and tobago" or trinidad or tobago or tunisia or turkey or turkmenistan or turkmen or uganda or ukraine or uruguay or uzbekistan or uzbek or vanuatu or "new hebrides" or venezuela or vietnam or "viet nam" or "middle east" or "west bank" or gaza or palestine or yemen or yugoslavia or zambia or zimbabwe or "northern rhodesia" or "global south" or "africa south of the sahara" or "sub-saharan africa" or "subsaharan africa" or "africa, central" or "central africa" or "africa, northern" or "north africa" or "northern africa" or magreb or maghrib or sahara or "africa, southern" or "southern africa" or "africa, eastern" or "east africa" or "eastern africa" or "africa, western" or "west africa" or "western africa" or "west indies" or "indian ocean islands" or caribbean or "central america" or "latin america" or "south and central america" or "south america" or "asia, central" or "central asia" or "asia, northern" or "north asia" or "northern asia" or "asia, southeastern" or "southeastern asia" or "south eastern asia" or "southeast asia" or "south east asia" or "asia, western" or "western asia" or "europe, eastern" or "east europe" or "eastern europe") OR de:(Africa OR "South America" OR "Latin America" OR asia) | 2,299,474 |
| 11 | title:("developing country" or "developing countries" or "developing nation*" or "developing population*" or "developing world" or "less developed countr*" or "less developed nation*" or "less developed population*" or "less developed world" or "lesser developed countr*" or "lesser developed nation*" or "lesser developed population*" or "lesser developed world" or "under developed countr*" or "under developed nation*" or "under developed population*" or "under developed world" or "underdeveloped countr*" or "underdeveloped nation*" or "underdeveloped population*" or "underdeveloped world" or "middle income countr*" or "middle income nation*" or "middle income population*" or "low income countr*" or "low income nation*" or "low income population*" or "lower income countr*" or "lower income nation*" or "lower income population*" or "underserved countr*" or "underserved nation*" or "underserved population*" or "underserved world" or "under served countr*" or "under served nation*" or "under served population*" or "under served world" or "deprived countr*" or "deprived nation*" or "deprived population*" or "deprived world" or "poor countr*" or "poor nation*" or "poor population*" or "poor world" or "poorer countr*" or "poorer nation*" or "poorer population*" or "poorer world" or "developing econom*" or "less developed econom*" or "lesser developed econom*" or "under developed econom*" or "underdeveloped econom*" or "middle income econom*" or "low income econom*" or "lower income econom*" or "low gdp" or "low gnp" or "low gross domestic" or "low gross national" or "lower gdp" or "lower gnp" or "lower gross domestic" or "lower gross national" or lmic or lmics or "third world" or "lami countr*" or "transitional countr*" or "emerging economies" or "emerging nation*") OR ab:("developing country" or "developing countries" or "developing nation*" or "developing population*" or "developing world" or "less developed countr*" or "less developed nation*" or "less developed population*" or "less developed world" or "lesser developed countr*" or "lesser developed nation*" or "lesser developed population*" or "lesser developed world" or "under developed countr*" or "under developed nation*" or "under developed population*" or "under developed world" or "underdeveloped countr*" or "underdeveloped nation*" or "underdeveloped population*" or "underdeveloped world" or "middle income countr*" or "middle income nation*" or "middle income population*" or "low income countr*" or "low income nation*" or "low income population*" or "lower income countr*" or "lower income nation*" or "lower income population*" or "underserved countr*" or "underserved nation*" or "underserved population*" or "underserved world" or "under served countr*" or "under served nation*" or "under served population*" or "under served world" or "deprived countr*" or "deprived nation*" or "deprived population*" or "deprived world" or "poor countr*" or "poor nation*" or "poor population*" or "poor world" or "poorer countr*" or "poorer nation*" or "poorer population*" or "poorer world" or "developing econom*" or "less developed econom*" or "lesser developed econom*" or "under developed econom*" or "underdeveloped econom*" or "middle income econom*" or "low income econom*" or "lower income econom*" or "low gdp" or "low gnp" or "low gross domestic" or "low gross national" or "lower gdp" or "lower gnp" or "lower gross domestic" or "lower gross national" or lmic or lmics or "third world" or "lami countr*" or "transitional countr*" or "emerging economies" or "emerging nation*") | 105,847 |
| 12 | #10 OR #11 | 2,349,734 |
| 13 | #1 AND #2 AND #9 AND #12 | 12,636 |

**Appendix 2. Screening Tool**

1. Is the study conducted in Low and Middle-Income Countries, as per the latest World Bank Classification?
   1. Yes, Include and SeeQ.2
   2. No, Exclude the country.
2. Does the study have an experimental or non-experimental design with the comparison group, instrumental variables and interrupted time series?
   1. Yes, Include and see Q3.
   2. No, Exclude on study design.
3. Does the study evaluate an intervention (policy, programme, project, or practice) or a review of evaluations of an intervention aimed at promoting climate-smart agricultural practices for land/soil, water and biodiversity management?
   1. Yes, Include and See Q. 4
   2. No, Exclude as Exclude on intervention.
4. Does the study analyze the effect of the intervention on measuring well-being, including measures of adaptive capacity and resilience, coping strategies and perceptions about resilience, agriculture outcomes (e.g., yield and income), and social outcomes (e.g., gender relations and time use) or outcomes which include knowledge, attitudes and practices, agency and ownership and control over assets.
   1. Yes, Include and See Q. 5
   2. No, Exclude as Exclude on the outcome.
5. Does the study report effect for women farmers and participants as well as differential effects for women versus men and other groups of differing advantage (e.g., socioeconomic status, landholding, education level) engaged in agriculture and food systems or engages program staff and community-level influencers (youth/elderly) for promoting climate-smart agricultural practices for land/soil, water and biodiversity management?
   1. Yes, Include
   2. No, Exclude on Population

**Appendix 3. Coding tool**

**Data extraction**

- Region
  - East Asia and the Pacific
  - Europe and Central Asia
  - Latin America and the Caribbean
  - The Middle East and North Africa
  - South Asia
  - Sub-Saharan Africa
- World Bank income group
  - Low income
  - Lower middle income
  - Upper middle income
- Country (specify)
- Study design
  - Randomised controlled trial
  - Quasi-experimental study
  - Regression discontinuity/interrupted time series
  - Propensity score matching
  - Difference-in-difference
  - Combinations of designs
  - Other (specify)
- The target group of intervention
  - Women only
  - Both men and women
  - Community influencers
  - Agriculture extension officers
  - Trainers
  - Other
- Equity dimensions
  - Urban
  - Rural
  - Low-income group
  - Medium income group
  - High-income group
  - Land ownership
- Intervention categories
  - Knowledge dissemination and capacity-building approaches
    - Social networking and peer learning
    - Information and communication technologies
    - Group and individual training and demonstration
    - Agriculture extension services
    - Farmer field schools or their modifications
  - Financial approaches, including credit and subsidies
    - Cash transfers, vouchers, matching grants
    - Insurance against loss
    - Risk management strategies
  - Institutional arrangements
    - Collectivization (e.g., farmer cooperatives and federations)
    - Contract farming
    - Land titling
    - Community infrastructure (e.g., dams for irrigation)
    - Other (specify)
  - Interventions to promote participation
    - Community-based natural resource management. (Natural resource management committees
    - Gender-responsive planning and budgeting
    - Incentives and motivation to participate
    - Others (specify)
    - Participatory action research
  - Behaviour and social change communication
    - Gender transformative, e.g., Representation/leadership of women in community based NRM committees, social and behavioral change campaigns
    - Gender-accommodating approaches, e.g., initiatives that seek to generate income for women
    - Intersectionality, e.g., GESI (gender equity and social inclusion) approach
- State the programme or project name; if there is no representation, then list the location (town or village)
- Intervention description (provide descriptive detail about the intervention)
- Intervention start date
- Intervention end date
- Follow up duration
- Duration of study
- Intervention delivered by.
  - Researcher/trainer
  - Implementing agency staff
  - External agency (survey firm)
  - Government
  - Agriculture extension workers
  - Not clear
- Scale of intervention
  - Regional level
  - National level
  - Intermediate level
  - Local level
  - Individual-level
- Outcome categories (code and add in info box how was each of the outcomes measured (what did they measure and how did they measure it?)
  - Intermediate outcomes
    - Increased knowledge and skills
    - Ownership and control of assets
    - Indicators of an agency such as decision-making and Connor- Davidson Resilience Scale (CD-RISC)
  - Endpoint outcomes
    - Coping mechanism strategies and risk mitigation strategies
    - Perceptions about resilience to shocks
    - Consumption smoothing capacity
    - Agricultural yield and income
    - Nutrition (e.g., height-for-age of children, body mass index of women and men)
    - Availability of time (time-use-workload/leisure)
- Comparison
  - No access or promotion of non- climate-smart technologies
  - Different in

Intervention than the promotion of CSA Intervention promoted with different intensity.

**Appendix 4. Risk of bias**

| **Bias domain** | **Question** | **Scoring criteria** | **Decision rules** |
| --- | --- | --- | --- |
| **1** | **1a. Confounding**: | Was the allocation or identification mechanism able to address confounding? | |
|  | **RCT** | **a) Sequence generation:** | - Score “Low risk” if all criterion are satisfied. |
|  |  | **-** The authors describe a random component in sequence generation/ randomisation method (e.g., lottery, coin toss, random number table). * | - Score "Some concerns" if there is no balance table reported (or key variables are omitted from the table) -- Score "High risk" if there is any failure in the allocation mechanism which could affect the randomisation process, or there is no balance table reported (c) and there is evidence suggesting a problem in the randomisation, such as covariate means are very different or sample size is too small for the procedure used (using stratification when there are less than two units for each intervention and control group in each strata can lead to imbalance), or if the paper does not provide details on the randomisation process or uses quasi-randomisation (e.g., alternate households allocated) which it is not clear has generated allocations equivalent to randomisation. |
|  |  | **-** If a special randomisation procedure is used to ensure balance, it is well described (stratification, pairwise matching, unique random draw, multiple random draws etc.) and adjustment is considered in the analysis (e.g., stratum fixed effects, pairwise matching variables). | * In order to assess the validity of the quasi-randomisation process, the most important aspect is whether the assignment process might generate a correlation between participation status and other factors (for example, gender, socio-economic status, pre-existing health condition) determining outcomes; consider whether assignment is done at cluster level (centralised) and covariate balance is reported. |
|  |  | **b) Subversion:** |  |
|  |  | - if the unit of allocation was by beneficiary or group, there was some form of centralised allocation mechanism such as an on-site computer system to ensure adequate allocation concealment. |  |
|  |  | **-** If a public lottery was used for the sequence generation, details were given on the exact settings and participants attending the lottery. |  |
|  |  | **c) Balance:** |  |
|  |  | **-** The unit of allocation is based on a sufficiently large sample size to equate groups on average. |  |
|  |  | **-** A balance table is reported for all subgroups receiving differential treatment, comparing means and standard deviations of variables, including cluster-level variables. |  |
|  | **NRS using statistical matching** | **a)** Information about the programme targeting criteria is known, presented in the paper, and used to justify the statistical approach. | -Score "Low risk” if all criteria are addressed. |
|  |  | **b)** Matching is done on pre-test (or time-invariant) characteristics, including the outcome measured at pre-test; matches are geographically local; the variables used to match are relevant (for example, demographic and socio-economic factors) to explain both participation and the outcome (so that there can be no evident differences across groups in variables that might explain outcomes); and, for cluster-assignment, authors control for external cluster-level factors that might confound the impact of the programme.* | -Score "Some concerns " if the selection into the programme was done according to clear targeting rules, which are used as matching variables, but there are imbalances remaining after matching. |
|  |  | **c)** With the exception of Kernel matching, the means of the individual covariates are demonstrated to be equated for treatment and comparison groups after matching. | -Score "High risk" if programme assignment was self-selected by participants and no baseline data are available to match the participants or groups, or |
|  |  |  | matching was done based on variables that are likely to be affected by the programme, or relevant variables are not included in the matching equation including cluster-level variables. |
|  |  |  | * Accounting for and matching on all relevant characteristics is usually only feasible when the programme allocation rule is known and there are no errors of targeting. There are different ways in which covariates can be considered. Observable differences across groups can be incorporated in the framework of a regression analysis (e.g., propensity-weighted least squares) or can be assessed by testing equality of means between groups. Differences in unobservable characteristics can be account for using double differences (DD), fixed effects (FE) or random effects (RE) where unobservables are time-invariant. |
|  | **NRS using double differences (DD), fixed effects (FE) or random effects (RE) analysis of panel data*** | **a)** Outcomes are measured at pre-test (before intervention) and post-test (after intervention) using the same approach. | -Score "Low risk” all criteria are addressed. |
|  |  | **b)** Examination of secular trends in outcomes shows parallel trends across treatment and comparison groups during periods prior to intervention. | -Score "Some concerns" if selection into the programme was done according to clear rules, and equal trends demonstrated, but baseline imbalances between groups remained. |
|  |  | **c)**The method is combined by well-conducted statistical matching done according to clear programme allocation rules (see above), and baseline imbalances, including in the outcome are shown to be small. | -Score "High risk " if equal trends are not reported, and programme allocation was due to participant self-selection, programme allocation was self-selected by participants and some relevant time-varying characteristics are not controlled, or insufficient details are provided, for example on testing the equal trends assumption or about cluster-level variables. |
|  |  | **d)** A comprehensive set of individual time-varying characteristics is controlled, including any cluster-level covariates that may affect the impact of the programme (e.g., rainfall). ** | * DD, FE and RE regression models are sometimes complemented with matching strategies. This combination approach is superior since it only uses in the estimation the common support region of the sample size, reducing the likelihood of existence of time-varying unobservable differences across groups affecting outcome of interest and removing biases arising from time-invariant unobservable characteristics. |
|  |  |  | ** Knowing allocation rules for the programme – or even whether the non-participants were individuals that refused to participate in the programme, as opposed to individuals that were not given the opportunity to participate in the programme – can help in the assessment of whether the covariates accounted for in the regression capture all the relevant characteristics that explain differences between treatment and comparison. |
|  | **1b. Confounding - justification** | Justification for coding decision (include a summary of justification for rating, mentioning your response to all sub-questions, cite relevant pages). | |
| **2** | **2a. Selection bias:**was any differential selection into the study adequately resolved? | **a) Follow-up data:**If the study design is prospective, follow-ups are recorded for all eligible participant units from recruitment onwards (i.e., prior to treatment). This is best shown using a participant flow diagram or reporting sufficient information to construct one. | -Score “Low risk” if all relevant criteria are satisfied. |
|  |  | **b) Participant identification:** where the unit of allocation in a prospective study was at group level (geographical/ social/ cluster unit), allocation was performed on all units at the start of the study, or participants and recruiters are blinded to allocation status, or awareness is unlikely to affect recruitment differentially (e.g., participants chosen randomly using a sampling frame based on census and response rate is high). | -Score “Some concerns” if the study used prospective design with adequate concealment, but no (or an incomplete) study flow diagram is reported, or in retrospective design where statistical methods are used to correct for selection bias. |
|  |  | **c) Balance:** a table is reported for all subgroups receiving differential treatment within control or treatment groups, comparing means and standard deviations of variables; any unbalanced covariates at individual level are controlled in adjusted analysis, including cluster-level variables. | -Score “High risk” if there are threats to adequate concealment (e.g., individual participants were chosen after cluster assignment was conducted or known, and there are differences between characteristics of the two groups beyond those expected by chance alone), or there is evidence of differential recruitment into study arms and differences in characteristics of groups not compatible with chance, or if no information is presented about participant characteristics or, in a prospective study, no study flow diagram (or data to construct it) presented. |
|  |  | **d) Selection bias analysis:**where evidence suggests there is selection bias into the study due to censoring of data (e.g., immortal time bias), this is accounted for using appropriate statistical methods (e.g., propensity weighted regression, Heckman selection model, proportional hazards model). |  |
|  | **2b. Selection bias - justification** | Justification for coding decision (include a summary of justification for rating, mentioning your response to all sub-questions, cite relevant pages). | |
| **3** | **3a. Attrition bias:** | **a) Attrition at cluster-level**is sufficiently low and similar reasons for attrition in treatment and control. Sufficiently low attrition is defined as: | -Score "Low risk" if overall attrition is less than 10 percent and differential attrition less than 10 percentage points at cluster (a) and individual (b) levels, and the study demonstrates robustness to attrition. |
|  | was any differential selection out of the study adequately resolved? | **-** total attrition (losses to follow-up) between pre-test and post-test in the study less than 10 percent of clusters (low risk) or 20 percent (some concerns). |  |
|  |  | **-** differential cluster attrition across study arms is less than 10 percentage points, and reasons for attrition are given and similar across groups. |  |
|  |  | **b) Attrition at individual-level**is sufficiently low and similar reasons for attrition in treatment and control. Sufficiently low attrition is defined as: | -Score "Some concerns" if overall attrition is between 10% and 20% and differential attrition less than 10 percentage points. |
|  |  | **-** total attrition (losses to follow-up) between pre-test and post-test in the study less than 10 percent of observations (low risk) or 20 percent (some concerns). |  |
|  |  | **-** differential attrition across study arms is less than 10 percentage points, and reasons for attrition are given and similar across groups. |  |
|  |  | **c) Robustness to attrition:**the study assesses losses to follow-up to be random draws from the sample (for example, by examining correlation with key characteristics across groups, or an F-test of attrition on baseline characteristics and interacted with treatment status), and study participants are randomly sampled. | -Score "High risk" if overall attrition exceeds 20% or differential attrition exceeds 10 percentage points, or there is some indication that the survey respondents were purposively sampled in a way that might have led the sampling to be different between treatment and control groups, or there is insufficient information on sampling methods, or no information on attrition is given. |
|  | **3b. Attrition bias - justification** | Justification for coding decision (include a brief summary of justification for rating, mentioning your response to all sub questions, cite relevant pages). | |
| **4** | **4a. Motivation bias:**was the process of observation free from motivation bias? | **Are criteria adequately addressed?** | -Score “Low risk” if either criterion is satisfied. |
|  |  | **a)**For data collected in the context of a particular intervention trial (randomised or non-randomised assignment), the authors state explicitly that the process of monitoring the intervention and outcome measurement is blinded to participants and outcome assessors, or methods are used that would minimise risk of Hawthorne effects, John Henry effects or survey effects such as infrequent observation or outcome questionnaires not referring to the intervention. Authors may also adapt the study design to estimate possible survey and Hawthorne effects (e.g., a ‘pure control’ with no monitoring except baseline endline). | -Score "Some concerns" if there was imbalance in the frequency of monitoring in intervention groups, which could have influenced behaviour in treatment and control differentially. |
|  |  | **b)** Informed consent is not associated with a particular intervention, as in the case of a regular household survey or a cluster-RCT, data are collected from administrative records, or in the context of a retrospective (*ex post*) evaluation. | - Score "High risk" if authors do not use an appropriate method to prevent possible motivation biases through blinding or other controls (e.g., infrequent measurement, methods to ensure consistent monitoring across groups, measurement using a ‘pure control’). |
|  | **4b. Motivation bias - justification** | Justification for coding decision (include a brief summary of justification for rating, mentioning your response to all sub questions, cite relevant pages). | |
| **5** | **5a. Performance bias:** | **a)** There were no implementation issues that might have led the control participants to receive the treatment, or authors use intention-to-treat (ITT) estimation. | -Score “Low risk” if all criteria are satisfied. |
|  | was the study adequately protected against spillovers, no-shows and crossovers? | **b)** The intervention is unlikely to spill over to comparisons (e.g., participants and non-participants are geographically and/or socially separated from one another and general equilibrium effects are not likely), or the potential effects of spillovers were measured (e.g., variation in the % of units within a cluster receiving the treatment). | -Score "Some concerns" if there is no obvious problem but there is no information reported on potential risks related to spillovers or contamination in the control group, or if there were issues with spillovers but they were controlled for or measured, or if any of the criteria are not satisfied but the scale of the issue is minimal. |
|  |  | **c)** There is no risk of substitution (differential contamination) by external programs (also called treatment confounding): participants are isolated from other interventions which might be received differentially between treatment and controls which could explain changes in outcomes. | -Score “High risk” if any of the criterion are not satisfied and happened at a large scale in the study, or if spillovers, no-shows, crossovers, implementation fidelity, or adherence to continuous interventions, are not reported clearly. |
|  |  | **d)** Errors in implementation fidelity by the intervening body were not systematic, or unlikely to affect the outcome. |  |
|  |  | **e)** For continuous interventions, measurement is taken of adherence to treatment among participants. |  |
|  | **5b. Deviation from interventions -justification** | Justification for coding decision (include a brief summary of justification for rating, mentioning your response to all sub questions, cite relevant pages). | |
| **6** | **6a. Measurement error:** | **a)** The study is a prospective design or in a retrospective design, participation in the intervention is observed, or the intervention clearly and consistently defined and misreporting by participants or enumerators is unlikely. | -Score “Low risk” if all criteria are satisfied. |
|  | is the study free from biases in measurement of intervention and outcomes? | **b)** Outcomes are clearly and consistently defined for all participants and outcome assessors in the study. | -Score "Some concerns" if there is a small risk related to any criteria and potential biases are measured, e.g., with placebo outcomes, and found to be null. |
|  |  | **c)** Outcomes are measured through observation (rather than self-report), and outcome assessors are blinded to intervention, or it is shown they are unbiased (e.g., spot-checks to validate). | -Score "high risk" if there are risks related to any criteria and authors were not able to control for the bias, or no information is provided to justify the absence of bias. |
|  |  | **d)** For self-reported outcomes: respondents in the intervention group are not more likely to report accurately than controls due to recall bias. |  |
|  |  | **e)** Respondents do not have incentives to over/under report something related to their performance or actions, or researchers put in place mechanisms to reduce the risk of reporting bias (irregular or infrequent data collection rounds, outcome assessors not involved in the implementation of the intervention, it is clear that answers to the survey will not affect what they receive in the future), or authors have measured bias through falsification tests (e.g., ‘placebo outcomes’ in cases where there was a risk of reporting bias). |  |
|  |  | **f)** Timing of the data collection did not differ between intervention and comparison group, the baseline data are not likely to be differentially affected by the time of intervention (e.g., due to seasonality). |  |
|  | **6b. Measurement error - justification** | Justification for coding decision (include a brief summary of justification for rating, mentioning your response to all sub questions, cite relevant pages). | |
| **7** | **7a. Analysis reporting bias: RCTs** | **a)** Authors report results corresponding to the outcomes announced in the method section (there is no outcome reporting bias). | -Score "Low risk" if all criteria are satisfied. |
|  | Was the study free from selective analysis reporting? | **b)** Authors report multiple analyses appropriately (e.g., by age group, sex). | -Score "Some concerns" if all the conditions are met except a), or if all the conditions are met but there is some element missing that could have helped understand the results better. |
|  |  | **c)** A pre-analysis plan or trial protocol is published and referred to or the trial was pre-registered, or the outcomes were pre-registered. | -Score "High risk" if no pre-analysis plan or trial protocol was published or pre-registered. |
|  |  | **d)** Authors report appropriate analysis methods, including results of unadjusted analysis and ITT estimation, alongside any adjusted and treatment-on-the-treated/complier-average-causal-effects analysis. |  |
|  |  | **e)** Analysts were blinded to treatment status. |  |
|  | **7b. Analysis reporting bias: NRS** | **a)** There is no evidence that outcomes were selectively reported (e.g., results for all relevant outcomes in the methods section are reported in the results section). | -Score “Low risk” if all criteria are satisfied. |
|  | Was the study free from selective analysis reporting? | **b)** Authors use credible methods of analysis to address attribution given available data. | -Score "Some concerns" if authors combined methods and reported relevant tests (d) only for one method, or if all the criteria are met except for c) and it is a retrospective NRS. |
|  |  | **c)** A pre-analysis plan is published, especially for prospective NRS (but ideally also for retrospective studies). | -Score "High risk" if authors use uncommon or less rigorous estimation methods such as failure to conduct multivariate analysis for outcomes equations, or if some important outcomes are subsequently omitted from the results or the significance and magnitude of important outcomes was not assessed. |
|  |  | **d)** Requirements for specific methods of analysis: |  |
|  |  | - For RDD, Researchers should analyze the change in slope and/or level using different band-widths around the threshold or functional form. The following should be pre-specified as far as possible and reported in sensitivity analysis: (a) selection of optimal bandwidth using existing data-driven routines; (b) selection of appropriate functional form for the relationship between assignment and outcome variables; and (c) robustness checks of other bandwidths and functional form specifications. |  |
|  |  | - For PSM and covariate matching: (a) Where over 10% of participants fail to be matched, sensitivity analysis is used to re-estimate results using different matching methods (Kernel Matching techniques); (b) For matching with replacement, no single observation in the control group is matched with a large number of observations in the treatment group, and authors take into account the use of control observations multiple times against the same treatment in the standard error calculation; (c) for PSM, Rosenbaum’s test suggests the results are not sensitive to the existence of hidden bias; (d) different matching methods including varying sample sizes yield the same results. |  |
|  |  | - For IV models, the authors test and report the results of a Hausman test for exogeneity (p≤0.05 is required to reject the null hypothesis of exogeneity). |  |
|  |  | - For Heckman selection models, the coefficient of the selectivity correction term (Rho) is significantly different from zero (p<0.05). |  |
|  | **7c. Analysis reporting bias - justification** | Justification for coding decision (include a summary of justification for rating, mentioning your response to all sub questions, cite relevant pages). | |

**Appendix 5. Possible candidate studies identified via a preliminary search**

| Title | Authors | Year | Study objectives | Country | Study design | Target group of intervention | Interventions | Outcomes | Inclusion? |
| --- | --- | --- | --- | --- | --- | --- | --- | --- | --- |
| Can Agricultural Input Subsidies Reduce the Gender Gap in Modern Maize Adoption? Evidence from Malawi | Fisher Monica ; Kandiwa Vongai ; |  | Impact of fertiliser subsidies on adoption of modern maize variety | Malawi | Instrumental variables estimation *simulations based on the logit adoption model* | Both men and women | Financial approaches including credit and subsidies *(Farm Input Subsidy Program (FISP)* | Agriculture yield and income | No |
| Adoption and Dissemination Pathways for Climate-Smart Agriculture Technologies and Practices for Climate-Resilient Livelihoods in Lushoto, Northeast Tanzania | Nyasimi Mary ; Kimeli Philip ; Sayula George ; Radeny Maren ; Kinyangi James ; Mungai Catherine ; | 2017 | This study is a follow-up of the FotF pilot in Tanzania and examines the effectiveness of the FotF as a mechanism for enhancing adaptation learning and identifies promising information dissemination pathways | Tanzania | Household survey data-quantitative effectiveness *focus group discussion-qualitative data* | Smallholder farmers Both men and women | Knowledge dissemination and capacity building approaches *(Farms of the Future Approach (FotF)* *approach-trigger farmers to learn, share, and adopt new climate-resilient agricultural practices and technologies) Fotf uses Climate Change, Agriculture and Food Security (CCAFS), a climate-analogue tool that can be used to connect sites with statistically similar climates (analogous) across space and/or time* | Increased knowledge and skills | Yes |
| Transforming Agricultural Extension Service Delivery through Innovative Bottom–Up Climate-Resilient Agribusiness Farmer Field Schools | Osumba Joab J L; Recha John W; Oroma George W; | 2021 | To improve the decision-making skills of implementors in the Climate-resilient agribusiness FFS (CRAFFS) approach, including the use of climate information to man- age climate-related risks that prevent farmers from closing yield gaps. The medium-term objective was to improve agricultural productivity, build resilience and achieve climate change mitigation and co-benefits where possible. The ultimate objective was to increase the capacity of actors to apply climate-smart technologies, practices and innovations, with the aim of increasing their adoption among farmers, agribusiness SMEs and farmer coop- eratives | Kenya, Tanzania and Uganda | Pre-post | Small holder farmers, both men and women | C*limate-resilient agribusiness Farmer Field School (CRAFFS)* | Knowledge and skill, perception about resilience to shock | Yes |
| Smart subsidies for sustainable soils: Evidence from a randomized controlled trial in southern Malawi | Ward Patrick S; Mapemba Lawrence ; Bell Andrew R; | 2021 | Evaluates a novel Payments for Ecosystem Services (PES) in the Shire River Basin in southern Malawi encouraging the adoption of CA | Malawi | RCT, treatment group further randomly allocated to one of two different payment treatments (conventional voucher or agglomeration payment) | Small holder farmers, both men and women | Financial approached including credit and subsidies | Increased knowledge and skill and coping mechanism strategies and risk mitigation, adoption of CSA | No |

[Enter text here]

**Appendix 6. Data extraction for Effect Size Calculation**

| 1. | Study id (EPPI ID) |
| --- | --- |
| 2. | First author surname and year |
| 3. | Which outcome is being coded (knowledge and skills-1; ownership and control of asses-2; indicator of agency-3; coping mechanism and risk mitigation-4; perception about resilience to shock-5; consumption smoothening capacity-6; agricultural yield and income-7; nutrition-8; time use-9 |
| 4. | Definition of outcome (provide authors description of outcome) |
| 5. | Follow up period (in months) |
| 6. | Sub-group analysis? (Yes-1; no-2) |
| 7. | Sub-group analysis description |
| 8. | Sub-group analysis description |
| 9. | Data to be extracted (continuous means and sd-1; dichotomous-proportions-2; regression-3 |
| 10. | Unit of analysis (individual farmers-1; group of farmers-2; Clusters (e.g villages)  -3; Others (specify)-4; not reported-5) |
| 11. | Treatment effect estimated (ITT-1; ATET-2; ATE_3; LATE-4) |
| 12. | **Baseline data** |
|  | Baseline Sample size (treatment group) |
|  | Baseline Sample size (control group) |
|  | Baseline Sample size (total) |
| 13. | **Endline data** |
|  | Endline Sample size (treatment group) |
|  | Endline Sample size (control group) |
|  | Endline Sample size (total) |
| 14. | **Outcome data (if continuous, mean and SD)-Baseline** |
|  | Baseline outcome treatment |
|  | SD baseline outcome treatment |
|  | Baseline outcome control |
|  | SD baseline outcome control |
| 15. | **Outcome data (if continuous, mean and SD)-post-intervention** |
|  | outcome treatment |
|  | SD outcome treatment |
|  | outcome control |
|  | SD outcome control |
| 16. | **Outcome data (if continuous, mean and SD)-follow up** |
|  | outcome treatment |
|  | SD outcome treatment |
|  | outcome control |
|  | SD outcome control |
| 17. | **Outcome data (mean difference and SD/SE at follow up** |
|  | Mean difference at follow up |
|  | SD at follow up |
|  | SE |
| 18. | **Outcome data if dichotomous (proportions)-Baseline** |
|  | Baseline number with outcome in treatment |

**Appendix 7. Risk of bias of included studies**

1. Dar M H, Waza S A; Nayak S and Chakravorty R ; Zaidi N W; Hossain M ;. (2020). Gender focused training and knowledge enhances the adoption of climate resilient seeds. *Technology in society*, 63, pp.101388.
2. Djido A, Zougmoré R B and Houessionon P ; Ouédraogo M ; Ouédraogo I ; Diouf N S;. (2021). To what extent do weather and climate information services drive the adoption of climate-smart agriculture practices in Ghana?. *Climate Risk Management*, 32, pp.100309.
3. Chowdhury Ataharul, Odame Helen Hambly and Thompson Shirley ; Hauser Michael ;. ( ). Enhancing farmers’ capacity for botanical pesticide innovation through video-mediated learning in Bangladesh. , 13(4), pp..
4. Guo Mingliang, Jia Xiangping and Huang Jikun ; Kumar Krishna B; Burger Nicholas E;. ( ). Farmer field school and farmer knowledge acquisition in rice production: Experimental evaluation in China. , 209, pp..
5. Mancini F. (2006). I*mpact of integrated pest management farmer field schools on health, farming systems, the environment, and livelihoods of cotton growers in Southern India*. . .
6. Nyasimi M, Kimeli P and Sayula G ; Radeny M ; Kinyangi J ; Mungai C ;. (2017). Adoption and Dissemination Pathways for Climate-Smart Agriculture Technologies and Practices for Climate-Resilient Livelihoods in Lushoto, Northeast Tanzania. *Climate*, 5(3), pp.63.
7. Osumba J J and Recha J W; Oroma G W;. (2021). Transforming Agricultural Extension Service Delivery through Innovative Bottom–Up Climate-Resilient Agribusiness Farmer Field Schools. *Farmer Field Schools. Sustainability*, 13(7), pp.3938.
8. Sharma P, Kaur L and Mittal R ; Kaur S ; Kaur S ;. (2019). Social marketing approach to bring change in water use behaviour of rural people of Punjab, India. *Journal of Water and Climate Change*, 10(4), pp.968-976.
